# Grape Berry Responses to Sequential Flooding and Heatwave Events: A Physiological, Transcriptional, and Metabolic Overview

**DOI:** 10.3390/plants11243574

**Published:** 2022-12-17

**Authors:** Alessandro Botton, Francesco Girardi, Benedetto Ruperti, Matteo Brilli, Veronica Tijero, Giulia Eccher, Francesca Populin, Elisabetta Schievano, Tobia Riello, Sergi Munné-Bosch, Monica Canton, Angela Rasori, Valerio Cardillo, Franco Meggio

**Affiliations:** 1 Department of Agronomy, Food, Natural Resources, Animals and Environment—DAFNAE, University of Padova, Agripolis, Viale dell’università 16, Legnaro, 35020 Padova, Italy; 2 Interdepartmental Research Centre for Viticulture and Enology—CIRVE, University of Padova, Via XXVIII Aprile 14, Conegliano, 31015 Treviso, Italy; 3 Department of Biosciences, University of Milan, Via Celoria 26, 20133 Milan, Italy; 4 Unit of Fruit Crop Genetics and Breeding, Research and Innovation Centre—CRI, Edmund Mach Foundation—FEM, Via E. Mach 1, San Michele all’Adige, 38098 Trento, Italy; 5 Department of Chemical Sciences, University of Padova, Via Marzolo 1, 35131 Padova, Italy; 6 Department of Evolutionary Biology, Ecology and Environmental Sciences, University of Barcelona, Diagonal 643, 08017 Barcelona, Spain

**Keywords:** *Vitis vinifera*, RNAseq, drought, heat stress, gene expression, hormone response, abscisic acid, metabolism

## Abstract

Grapevine cultivation, such as the whole horticulture, is currently challenged by several factors, among which the extreme weather events occurring under the climate change scenario are the most relevant. Within this context, the present study aims at characterizing at the berry level the physiological response of *Vitis vinifera* cv. Sauvignon Blanc to sequential stresses simulated under a semi-controlled environment: flooding at bud-break followed by multiple summer stress (drought plus heatwave) occurring at pre-vèraison. Transcriptomic and metabolomic assessments were performed through RNASeq and NMR, respectively. A comprehensive hormone profiling was also carried out. Results pointed out a different response to the heatwave in the two situations. Flooding caused a developmental advance, determining a different physiological background in the berry, thus affecting its response to the summer stress at both transcriptional levels, with the upregulation of genes involved in oxidative stress responses, and metabolic level, with the increase in osmoprotectants, such as proline and other amino acids. In conclusion, sequential stress, including a flooding event at bud-break followed by a summer heatwave, may impact phenological development and berry ripening, with possible consequences on berry and wine quality. A berry physiological model is presented that may support the development of sustainable vineyard management solutions to improve the water use efficiency and adaptation capacity of actual viticultural systems to future scenarios.

## 1. Introduction

Grapevine (spp. *Vitis vinifera*) is a crop of high economic importance due to its wide distribution in the temperate zones of both hemispheres [1]. Nowadays, however, viticulture is facing several challenges, such as the reduction in water availability [2,3] and pesticide consumption [4], environmental sustainability [5], pathogens [6], and, last but not least, climate change and global warming [7,8,9,10]. In addition to the progressive increase in global temperature, IPCC (Intergovernmental Panel on Climate Change) also included “*extreme events*”, such as floodings, heatwaves, heavy hail, and prolonged dry spells, among the “*key risks*” (IPCC, 2022; [11]), defining an extreme weather event as “*an event that is rare at a particular place and time of year*”. The definition of “*rare*” is variable, but an extreme event should normally be “*rare*” or “*rarer*” than the “*tenth or ninetieth percentile*” (IPCC, 2014; [12]). In addition to the increasing frequency of such extreme weather events, there is also compelling evidence that those stresses may occur simultaneously [13], and sequential stress, such as spring flooding followed by multiple summer stresses, i.e., drought and heat waves, may occur quite commonly. Therefore, the current challenge is not only to understand how grapevine responds to a single stress but also if there are interactions among different stresses that may occur sequentially during the same productive season in order to find solutions other than moving the cultivation areas.

The optimal growth temperature for grapes is referred to as the temperature at which the plant reaches the optimum in terms of development, photosynthesis, and maturation, and it is generally located below 30 °C. If the ambient temperature exceeds 5 °C to this threshold, we can then speak of heat stress [14]. Temperatures above 40 °C can lead to inhibition of photosynthesis [15], while high temperatures above 45 °C may cause severe damage to the photosynthetic system and stomatal closure, and, if these conditions persist for a prolonged period, leaves may be irreversibly damaged and die [16]. According to Carbonneau [17], micro- and meso-climate characteristics are crucial for wine quality, so the long-term consequence of increased temperatures will be the displacement of the ideal area of cultivation of a certain variety, which will no longer express its maximum quality in the native terroir [18]. In a warmer climate, phenology is anticipated in terms of bud burst, flowering, véraison and ripening [19]; sugars accumulate faster and at higher levels [20], and acidity decreases due to higher respiration rates of organic acids caused by high temperatures, thus contributing to grapes with unbalanced sugar-to-total acidity ratios at harvest [21,22]. Consequently, the alcohol content of wines will increase, as already demonstrated both for Riesling produced in Alsace in the last thirty years [23] and for several Napa Valley wines [24]. Behind these measurable reactions to excessive heat, there are several physiological mechanisms driven by changes in the regulation of numerous cellular processes within the berry, among which the changes in gene expression are pivotal. Target gene expression analyses pointed out the up-regulation of *VvGOLS1*, a grape gene encoding a galactinol synthase, as a response to high temperatures applied locally to the grape clusters of the cv Cabernet Sauvignon [25]. Massive gene expression analysis of berries of the cv Muscat Hamburg undergoing heat stress pointed out the induction of *HSP* (Heat Shock Proteins) and *HSF* (Heat Shock Factors) genes, which may allow berry ripening progression through a stabilization of protein functions and transmembrane carriers [26]. Rienth et al. [27] observed different responses to heat stress according to the time of the day, especially for genes involved in organic acid and phenylpropanoid metabolisms in microvine plants. Microarray analyses carried out on the cv Cabernet Sauvignon pointed out that the most affected processes in the berry response to heat stress belong to the functional categories “stress responses”, “protein metabolism” and “secondary metabolism”, highlighting the intrinsic capacity of the berry to build adaptive responses upon heat stress perception [28]. More recently, Ye et al. [29] proposed that *VvBAP1*, coding for a grape C2 Domain Protein, may play a potential important role in enhanced grapevine thermoresistance, mainly by enhancing the expression of genes encoding antioxidant enzymes and other heat stress-responsive genes. Another recent study pointed out that the genes belonging to chaperone-mediated protein folding and cell-wall modifications were found to play a pivotal role in response to the heat of the grape cv Fantasy Seedless [30].

Grapevine can tolerate moderate to severe water shortage [31]. Stomatal regulation is one of the first processes to be triggered by the decrease in water potential, tending to close due to the increased local synthesis of abscisic acid (ABA) [32]. The closure of the stomata then causes a reduction in water transpiration, gas exchange, and, consequently, photosynthesis. Under long-lasting drought, cellular respiration is also inhibited. From the point of view of the gene expression changes occurring in the berry as a response to drought, most of the studies were performed on red grape cultivars, for which water stress is usually considered beneficial in stimulating anthocyanin biosynthesis at the transcriptional level [33]. Recent findings also demonstrated a cultivar-specific transcriptional response to drought [34], so a careful evaluation of the data available in the literature should be performed taking into account the specific genotype. Regarding white grapes, a wide metabolomic and transcriptomic study pointed out a list of 4889 genes that are differentially expressed at different times after water stress imposition, dealing with functions such as “carbohydrate metabolic process”, “development”, and “response to biotic stress” for the up-regulated genes, and “response to stress”, “transport”, and “response to abiotic stress” for the down-regulated [35]. In the same research, the expression of genes coding for enzymatic elements of the phenylpropanoid, terpenoid, and carotenoid pathways was shown to be significantly modulated by water deficit, as also demonstrated by Zombardo et al. [36] and Alatzas et al. [37]. Moreover, a putative pivotal role for the gene *VviMYB24* in controlling the monoterpene and fatty acid biosynthetic pathways in grapevine during abiotic stress response was also pointed out [35]. Regarding the hormonal response of the berry to drought, both ABA-dependent and ABA-independent mechanisms were shown to be involved. The latter being represented mainly by ethylene signaling elements (reviewed by Gambetta et al. [31]).

All the studies previously described were focused on the reactions of grape berries to isolated stress events, although vineyards are rarely exposed to single stresses. Indeed, heat and drought stress often occur simultaneously, and their combination is not necessarily equal to the sum of the effects of individual stresses. Recent studies carried out on several crops and model species concluded that each combination of stress triggers a specific set of responses in the plant [13,38]. For example, the combination of thermal and water stress results in the inhibition of photosynthesis and the accumulation of terminal products of lipid peroxidation [39,40]. At the transcriptional level, in Arabidopsis, several transcripts are over-expressed under single stresses and under-expressed when exposed to a combination of the same stresses and vice versa. For example, genes encoding various oxidase enzymes, PAL, WRKY transcription factors, as well as various pathogenesis-related proteins, seem to be activated during a combination of thermal and water stress. Under a single stress condition, on the other hand, the activated transcripts are, for example, dehydrins and catalases in the case of water stress, while in the case of thermal stress, peroxidases are prevalent [41]. Under combined stress, photosynthesis is markedly reduced compared to control and even more than under water stress alone, with the damage to Photosystem II depending on the species [42]. The most difficult challenge for the plant is, therefore, to balance the closing effect of the stomata caused by water stress with the opening signal stimulated by heat. In grapevine, under this stress combination, the influence of drought stress was shown to be prevalent [43]. In the same study, carried out on leaves, ABA and proline were shown to be good markers for drought and combined stress, respectively, and the transcription of key genes of their biosynthesis, i.e., *VvNCED1* and *P5CS*, displayed a good correlation with the levels of the two metabolites. Surprisingly, no transcriptomic study was found in the literature regarding the response to drought/heat combined stress in grape berries, except the research, carried out by Upadhyay and Upadhyay [30] regarding a transcriptome analysis of heat stress response of grape variety ‘Fantasy Seedless’ under different irrigation rates, not including, however, an actual water stress condition.

According to the most recent report of IPCC (2022), the frequency of extreme compound events such as pluvial flooding along with droughts and heat waves has increased in all parts of the world since the 1950s, and these events are included among the combinations of future changes of climatic impact-drivers in the Mediterranean area. Therefore, the areas where grapevine cultivation is more widespread are particularly challenged by such events, but little is known about the effects on grapevine physiology when a combination of these events occurs. Our study is placed in this scenario, which is common to most of the traditional wine growing regions, and aims at investigating for the first time at the physiological, molecular (gene expression), and metabolic level, the response of the berry to a combined sequence of spring (bud-break; BBCH09 according to Lorenz [44] flooding followed by summer (pre-véraison; BBCH79-81) heatwave, both simulated under semi-controlled conditions (thermostatic tunnels). The berry fruit response was assessed in both single- and double-stressed vines with respect to a non-stressed control. The cultivar Sauvignon Blanc, whose quality profile is significantly affected by different types of stress [45,46,47], was chosen in this study for being one of the most widespread in the world, especially in temperate areas where the above-reported climate change scenario is already impacting viticulture, and because most of the research on stress was previously carried out mainly on red grape varieties.

## 2. Results and Discussion

### 2.1. Experimental Conditions and Ripening Kinetics

**Figure 1:**
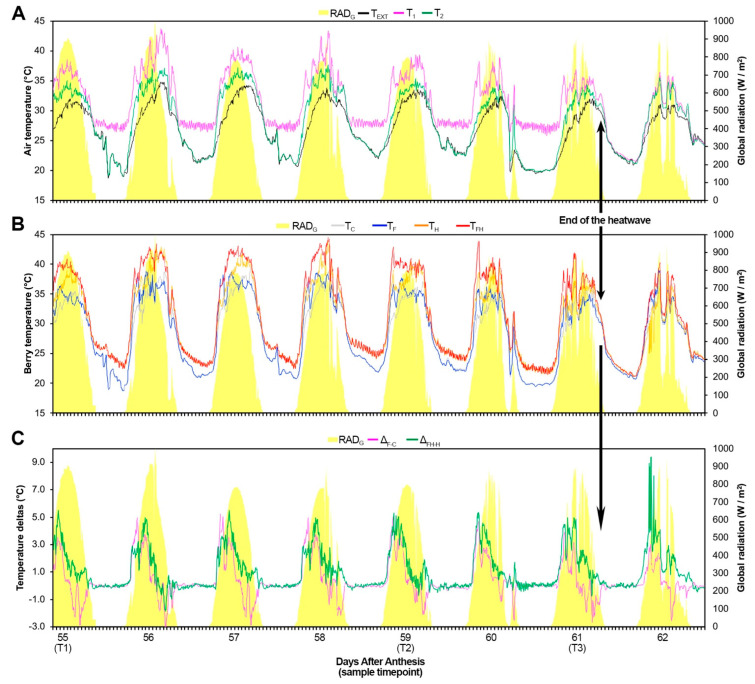
(**A**) Global radiation (RAD_G_) and air temperature measured outdoor (T_EXT_) at the site of the experiment (Legnaro, Padova, Italy; 45°20′58.8″ N 11°56′59.5″ E) and inside the tunnels used for the experiment (T_1_, Tunnel no.1; T_2_, Tunnel no.2). (**B**) Global radiation (RAD_G_) and berry temperature (T_C_, control; T_F_, spring flooding; T_H_, summer heatwave; T_FH_, spring flooding + summer heatwave). (**C**) Differences (deltas) of the temperatures of F and FH berries with respect to their control C (Δ_F—C_) and H (Δ_FH—H_), respectively, are shown along with the global radiation to give an idea of the day-night rhythmicity. All parameters shown in both charts were measured from the beginning of the second phase of the heatwave when temperature increased (T1, 55 DAA) up to one day (62 DAA) after the end of the heatwave, as indicated by the black arrows. The small oscillations observed in the temperatures measured in Tunnel no.1 were due to the histeresis of the thermostat that controlled the heating system.

With respect to the outdoor conditions, internal temperatures in both tunnels before heat wave induction was about 2.2 °C higher during the day and equal during the night (Figure 1). When the heatwave started 55 Days After Anthesis, DAA (T1), the air within Tunnel no.1 (containing the H and FH grapevines) was 3.3 °C and 5 °C warmer than Tunnel no.2 during the day and night, respectively. During the experiment, five thermocouples were inserted into the berries of five different plants for each treatment in order to measure the effective temperatures experienced by the berries under different treatments. In Tunnel no.2, the F berries were 0.5 °C warmer than the C ones during the day, while during the night, their temperatures were almost identical. In Tunnel no.1, the FH berries were 1.5 °C warmer than the H ones, while their temperatures were closely similar during the night. A comparison between the temperatures of the berries in the different tunnels indicated that the H berries were 2.5 °C and 2.8 °C warmer than the C ones, while the FH was 3.4 °C and 2.8 °C warmer than the F during the day and night, respectively. Just to give an idea of the entity of the double stress (flooding plus the heatwave), the FH berries were 4 °C and 2.8 °C warmer than the control ones. The higher temperatures observed in both F and FH berries with respect to C and H, respectively, occurred mostly during the morning. Before T2, when temperatures were increased, the temperature deltas of F berries with respect to C reached their maximum before the temperature delta of FH berries with respect to the H ones. After T2, the FH berries got warmer such as the F ones, in early morning, and interestingly, peaking with similar trends despite the absence of the heatwave induction in Tunnel no.2. In the afternoon, on the other hand, while the FH berries maintained warmer temperatures than the H ones, although, to a lesser extent, the F berries were even cooler than the C ones. Therefore, in the non-stressed Tunnel no.2, F berries got warmer than the C before and cooler later, while in the stressed Tunnel no.1 FH berries were always warmer than the H, reaching the maximum peak earlier before temperatures increased. Finally, on the day after the removal of stress conditions (62 DAA), FH berries reached their maximum difference of temperature with respect to the H ones, with deltas of about 9 °C in the early morning. Possible explanations for the warmer temperatures reached by berries that underwent the spring flooding were initially searched in either their size (small berries warm up faster than big ones) or their content of solutes (low solutes content implies less time to warm up than a high concentration), which could eventually justify a faster heating rate. However, from the measurements carried out during the experimental period, only the F berries resulted in being significantly smaller than the C ones and only at T2. On the other dates during the heatwave, differences were statistically non-significant (Figure S1). Regarding the solute content of the berries, as discussed below, F and FH berries showed higher values than C and H of Soluble Solids Content (SSC), thus not justifying their warmer temperatures. Moreover, it is also worth noting that the double-stressed berries seemed to keep a sort of memory and got even warmer after stress removal. In the next section, we compared the differentially expressed genes (DEGs) in common between the two contrasts F vs. C and FH vs. H and made an enrichment analysis; this was possible only at T1, as in T2 and T3, there were no DEGs in the contrast F vs. C, i.e., they were transcriptionally identical (statistically speaking) although the berries showed different temperatures. At T1, there were 116 genes upregulated and 131 downregulated in common between the contrasts F vs. C and FH vs. H. Only the former subset was enriched by GO terms, which dealt with plastid and thylakoid. These enriched gene functions which are active regardless of the stress imposition at T1, maybe just a consequence of the higher temperatures occurring in those berries, to which the parenchymatic fruit cells respond by changing the composition of thylakoid membrane lipids and proteins, as previously observed in both grapevine tissues [19,48] and other species [49].

**Figure 2:**
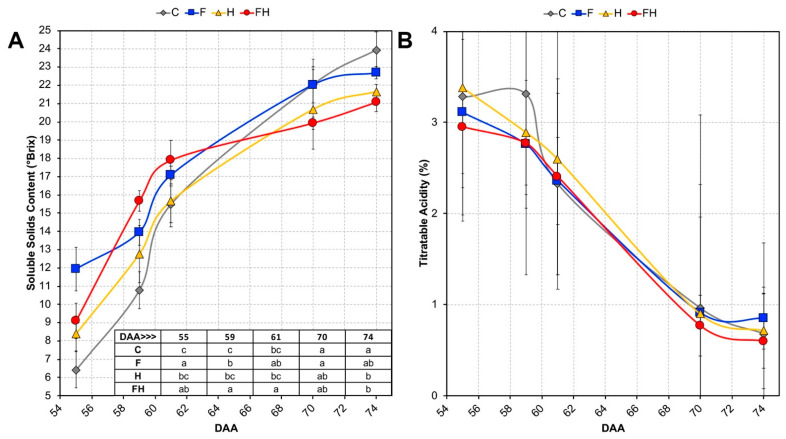
Soluble Solids Content (SSC; (**A**) and Titratable Acidity (**B**) were measured in 10 berries of each treatment, starting from the day when temperatures were increased (T1, 55 DAA) up to the end of the experiment, when berries were harvested (T5, 74 DAA). Bars represent standard error. Statistics were reported only for SSC with different letters marking statistically significant differences, while for titratable acidity, differences were all non-significant (*p* ≤ 0.05). (C, control; F, flooded; H, heatwave; FH, flooded plus heatwave).

The ripening kinetics of the berries were monitored starting from 55 DAA, corresponding to T1, by measuring both SSC and titratable acidity. Concerning the latter, no significant difference was pointed out among the different treatments, although slightly lower values were initially observed in the F and FH samples, then rapidly reached by the other samples keeping a substantial overlapping profile until T5 (Figure 2). As far as the SSC is concerned, interesting trends were pointed out as related to both the spring stress and the combined summer stress. Indeed, the F and FH samples showed higher SSC values at T1 up to T3, then FH and H berries joined together at T4, keeping lower values than the unstressed samples until T5. Taken together, these data indicate that berries that underwent the spring stress (F and FH) had a slight ripening advance with respect to the C and H samples until T3, then the effects of the heatwave realigned the behaviors of FH and H, preventing them from reaching the same SSC values of the non-stressed berries at harvest (T5). Since after véraison the transpiration rate of the berry progressively decreases [50], possibly because the stomata either become partially or completely occluded by epicuticular wax or are transformed into lenticels [51], the ripening advance observed in F and FH berries, which was also confirmed by gene expression data (see sections below), may have negatively affected their thermoregulatory ability, thus causing those berries to warm up faster than C and H.

### 2.2. RNASeq Analyses

RNA samples from each timepoint and treatment were sequenced, allowing us to obtain, on average more than 24 million total reads per sample (Table S1), of which about 90% were mapped, with approximately 600,000 reads (~3%) showing multiple alignments.

RNASeq data were validated through quantitative real-time PCR (qPCR) on a subset of fourteen genes chosen among the DEGs based upon both their annotations, i.e., the presence of GO terms resulted in being significantly enriched, mainly focusing on the T3 contrasts. A high correlation, as high as 0.77 (Pearson coefficient, *𝛒*), was pointed out between the RNAseq and the qPCR, with the main differential expression patterns fully matching with both techniques (Figure S2). This technical validation allowed us to consider the RNASeq data reliable in order to carry out functional inferences based on the trends of expression and enrichment analyses.

**Figure 3:**
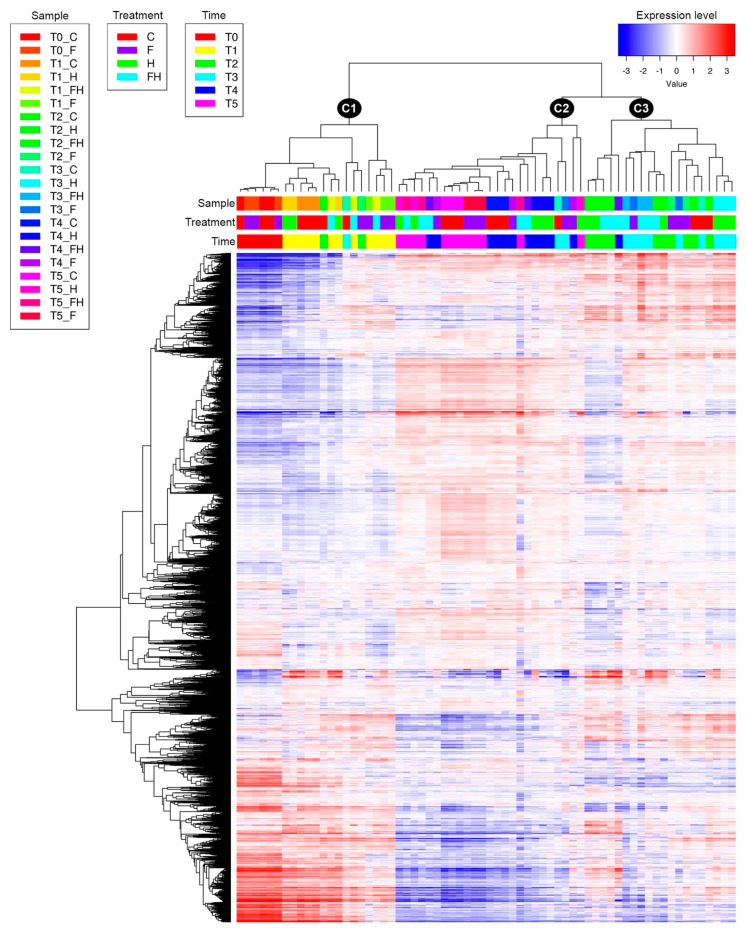
Heatmaps of the 5000 most variable genes and hierarchical clustering for both samples (columns) and genes (rows). The different replicates were marked at the top of the heatmap with different colors according to the sample to which they belong, the treatment (C, control; F, flooded; H, heatwave; FH, flooded plus heatwave), and the time of sample collection (a legend is provided on the top left). The three main clusters of samples (C1, C2, and C3) are indicated, and the scale of expression level is shown at the top right.

**Figure 4:**
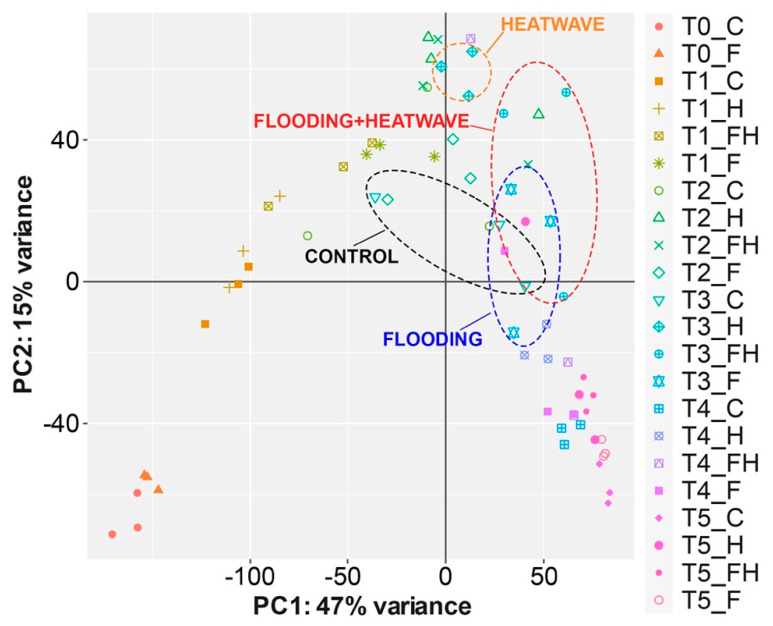
Principal component analysis of the samples built using the 5000 most variable genes. The ellipses show the most relevant group samples at T3 when the stress is maximum. The legend on the right provides indications of the colors and symbols used in the chart for each sample. Samples are named according to the timepoint and treatment (C, control; F, flooded; H, heatwave; FH, flooded plus heatwave).

By using the web-based tool iDEP (integrated Differential Expression and Pathway analysis [52], several exploratory analyses were carried out. Figure 3 shows the heatmap of the 5000 most variable genes along with the corresponding hierarchical clustering of samples and genes. One clade (C1) includes all the samples at T0 and T1 and also three single replicates of other timepoints (one replicate of T2 control, one of T2 flooding, and one of T3 control). The other big cluster is subdivided into two main subgroups, one (C2) with T4-T5 samples and two replicates of T3 samples (one from a control—C- and another one from flooding—F- sample), and another subgroup (C3) with T2-T3 samples and just one T4 replicate of an FH (flood + heatwave, i.e., the summer stress) sample. T0-T1 cluster shows contrasting expression levels compared to the other two subgroups, especially C2, and the proportion of genes with increasing expression during the experiment was much higher than those with decreasing trends. These gene expression changes and their general trend might be the result of the ripening progression in all the berries, indicating that ongoing development is stronger than any other factor, such as the stress imposed on the berries. In addition, a Principal Component Analysis (PCA; Figure 4) was also performed to identify the main factor affecting the variability of berries’ transcriptomes. The first two principal components were able to display 62% of the total variance between all berry samples. A negative parabolic tendency was observed in the plot, starting from T0 to T5. Samples were seemingly paired in the cluster by timepoints, being T0, T2-T3, and T4-T5 as the closest clusters, while samples from T1 were dispersed, as well as some samples from T2 and from T3. It is noteworthy that the samples at T3 that undergone the maximum summer stress alone (H), i.e., the heatwave (drought plus increased temperatures), grouped closer to each other than all the other treatments, thus indicating a likely strong and almost univocal transcriptional response in all the replicates.

Following the exploratory data, the DESeq2 package allowed us to identify the DEGs for each comparison by using both a false discovery rate (FDR) threshold equal to 0.05 and a fold-change (FC) threshold of 1.5. The main comparisons between treatments were: (1) C vs. F, (2) C vs. H, (3) C vs. FH, (4) H vs. F, (5) H vs. FH, and (6) FH vs. F, each one analyzed by its timepoint: T0, T1, T2, T3, T4, and T5. A complete list of these DEGs are reported in Table S2. Please note that in the following paragraphs, when a pattern of either up- or down-regulation will be described, we will generally refer to the first sample of the contrast: for example, if the contrast is C versus F, genes are either up- or down-regulated in C with respect to F.

**Figure 5:**
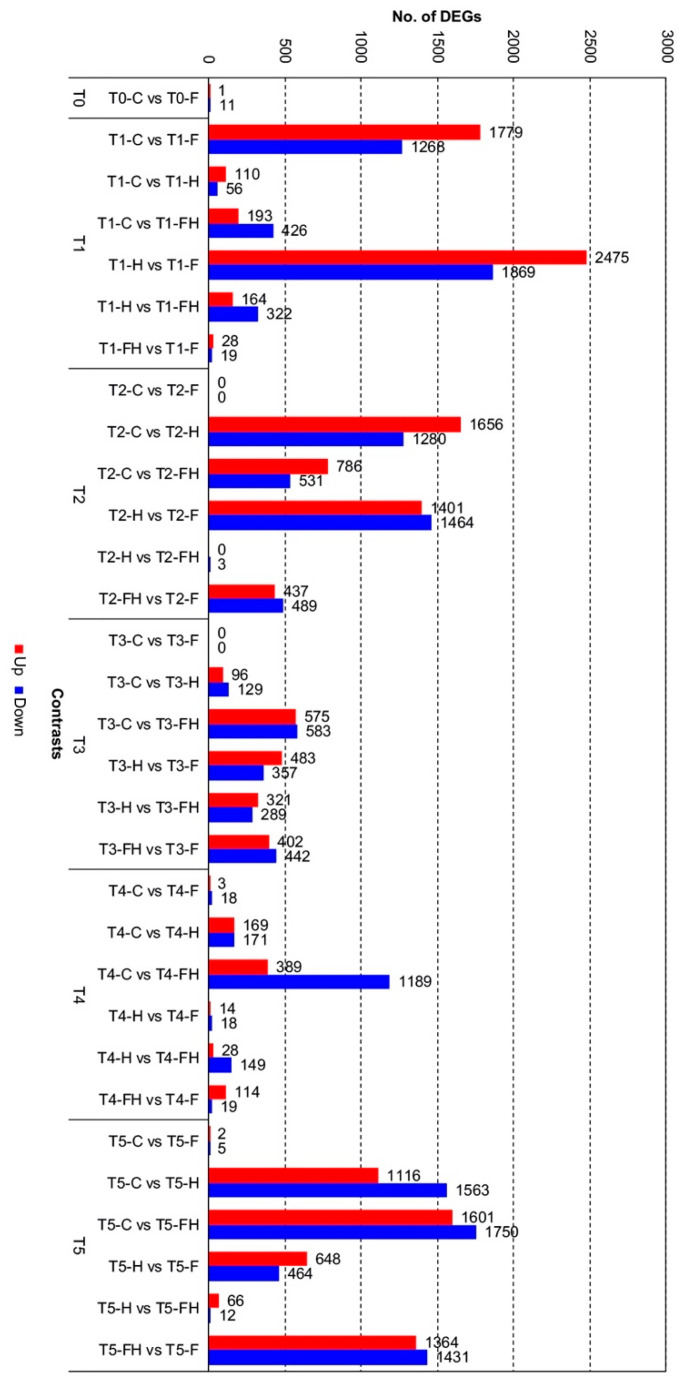
The number of differentially expressed genes (DEGs) in a selected number of contrasts from T0 to T5. Up- (red) and down- (blue) regulated genes are shown for each comparison. Samples within each contrast are named according to the timepoint and treatment (C, control; F, flooded; H, heatwave; FH, flooded plus heatwave).

A total of 12 DEGs were found between C and F at T0 (Figure 5), indicating just some weak differences between samples due to the flooding that occurred in spring. No significant GO terms functional enrichment was found among these genes. Similarly, at the following timepoints with the only relevant exception of T1, C berries versus those of the flooded plants (F) did not show any relevant number of DEGs and, eventually, no enrichment by GO terms was found except for the comparison at T4 (see below).

**Figure 6:**
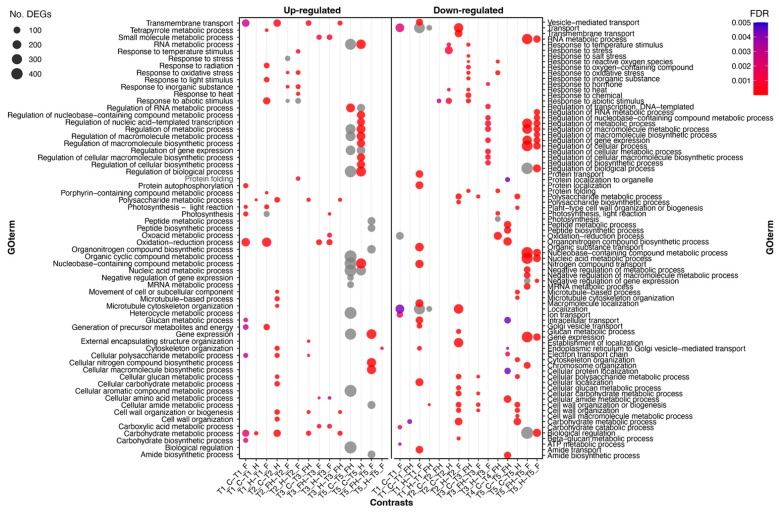
Dot plot of the enriched GO terms in the different contrasts. For each GO term enriched in either up-regulated (**left**) or down-regulated genes (**right**), the number of DEGs annotated, and the FDR statistics are represented as shown in the legends at the top left and right, respectively. Samples within each contrast are named according to the timepoint and treatment (C, control; F, flooded; H, heatwave; FH, flooded plus heatwave).

Concerning T1, in contrast, C versus F berries, a total of 3047 DEGs were identified, of which 1779 were up-regulated and 1268 down-regulated in C samples (Figure 5). The most significant enriched terms in this contrast were, among the most interesting, the ones related to the up-regulation of “*Photosynthesis*”, followed by “*Protein autophosphorylation*”, “*Oxidation-reduction process*”, “*Carbohydrate biosynthetic process*”, and “*Auxin activated signaling pathway*”, whereas among the down-regulated terms were “*Carbohydrate catabolic process*”, “*Transport*”, “*ATP metabolic process*”, and “*Localization*” (Figure 6). The genes annotated with the GO term related to auxin signaling may provide a reliable explanation for the high number of DEGS found between these two T1 samples. It is noteworthy that among these transcripts, which were up-regulated in C berries (and thus down-regulated in the F berries), there are six putative *ARFs* (Auxin Response Factors; *VIT_202S0025G01740, VIT_206S0004G03130, VIT_208S0040G01810, VIT_210S0003G04100, VIT_213S0019G04380, VIT_215S0046G00290*), four *Aux/IAAs* (*VIT_205S0020G04680, VIT_205S0020G04690, VIT_214S0030G02310, VIT_218S0001G08090*), two *Auxin Efflux Carriers* (*VIT_217S0000G02420, VIT_214S0006G01970*), two *Auxin Induced Proteins* (*VIT_205S0049G01970, VIT_207S0005G04380*), and two *Phytochrome Associated Proteins* (*VIT_209S0002G04080, VIT_211S0016G03540*) previously shown to be down-regulated at véraison [53]. Considering the data shown in Figure 2 regarding the Soluble Solids Content, this would indicate that the high number of DEGs found in this T1 contrast may be due to an advanced ripening stage in berries of flooded grapevines, presumably related to the waterlogging stress that occurred in spring. For the berries undergoing the heatwave, the highest number of DEGs was found between H and F at T1, with 2475 genes up-regulated and 1869 down-regulated, in which the GO term “*Photosynthesis*” was the most significantly enriched for the up-regulated genes, while the down-regulated genes presented a highly significant enrichment in “*Localization*”, “*Transport*”, and “*Vesicle-mediated transport*” GO terms. Among these DEGs, 1311 and 929 are either up- or down-regulated also in contrast between C and F, thus leading to hypothesize that most of the differences are due to the slight ripening advance caused by spring flooding. Opposite to this result, the comparison between FH and F showed no significant enrichment as the number of DEGs was quite low (47 in total), thus indicating a possibly weak (or delayed) response to the initial stress conditions in the berries coming from grapevines previously subjected to flooding. At T1, also the comparison between C and H did not give high numbers of DEGs (166 in total), whereas the other two contrasts (C vs. FH and H vs. FH) gave higher numbers (619 and 486, respectively) possibly because flooded sample was involved in the comparison, thus biasing the results due to the slight advance in the ripening of the berries. This is also supported by the enriched GO terms found in these contrasts, most of which deal with cell wall polysaccharide metabolism that may mark ripening inception (Figure 6).

Within the six contrasts at T2, the comparison between C and H berries presented a total of 2936 DEGs, 1656 genes up-regulated and 1280 down-regulated, followed by the contrast between H and F with a total of 2865 DEGs. Among the 1401 and 1464 genes, either up- or down-regulated in the latter, 936 and 1134 were in common with those of the previous comparison, exactly with the same regulatory pattern. In both cases, DEGs were enriched by GO terms related to eleven common functions, i.e., “*Carbohydrate metabolic process*”, “*Polysaccharide metabolic process*”, “*Cellular polysaccharide metabolic process*”, “*Cell wall organization or biogenesis*”, “*Cellular glucan metabolic process*”,“*Cellular carbohydrate metabolic process*”, “*Cell wall organization*”, “*Transmembrane transport*”, “*Response to heat*”, “*Response to temperature stimulus*”, and “*Response to abiotic stimulus*”. In addition these functions commonly regulated in the two contrasts, there were also some contrast-specific GO term enrichments, which can be useful to discriminate the different responses to the heatwave in the plants previously flooded (F) with respect to the control ones (C). For example, with respect to the flooded plants, the heatwave repressed the expression of genes dealing with “*Transport” and “Localization*” and increased the number of transcripts dealing with “*Response to oxidative stress*”, “*Response to hydrogen peroxide*”, “*Response to reactive oxygen species*”, and “*Glutathione metabolic processes*”. Comparing the combined effect of the summer stress taking the control plants as a reference pointed out the upregulation of genes functionally involved in “*Microtubule-based process*” and “*Cytoskeleton organization*”. These results evidenced a differential functional response to the heatwave in the grapes that underwent the spring stress due to waterlogging with respect to the control ones. It is worth noting, however, that in the comparison between F and FH, there were just three DEGs. If we compare the enrichment analyses of previous contrasts with those coming from the DEGs between FH versus F (926 DEGs) and C versus FH (1317 DEGs), most of the GO terms dealing with oxidative stress and response to high temperatures are in common. In the latter comparison (C versus FH), however, there are also some specific enriched functions linked to trehalose, such as for the genes *VIT_201S0011G05960* (coding for a UDP-Glycosyltransferase/trehalose-phosphatase family protein), *VIT_201S0026G00280* (coding for a trehalose phosphate synthase), *VIT_210S0003G01680* and *VIT_217S0000G08010* (both encoding a trehalose-phosphatase/synthase). All these genes were strongly and significantly upregulated at T2 in stressed berries that did not undergo spring flooding.

At the end of the heatwave (T3), at the maximum level of stress, the general situation seemed to be much more stable than before, at least in terms of the number of DEGs, although with a few exceptions. While in the C/H and H/F contrasts, the number of DEGs dropped to 225 and 840, respectively, in the C versus FH, there were still 1158 DEGs. In the first two comparisons, functional annotation is noteworthy that deals with the “*Hormone-mediated signaling pathway*” present in eight genes commonly regulated, among which there are three genes linked to auxin (*VIT_217S0000G02420*, *VIT_210S0003G00420, VIT_215S0046G01280*) that are down-regulated by the heat stress. The DEGs in the C/FH contrast were enriched by functions dealing with response oxidative stress (up-regulated in the stressed berries), indicating an ongoing active reaction of the berries to the maximum stress, and cell wall biogenesis (down-regulated in the stressed berries), still indicating a state of strong sufferance. The latter functional enrichment is strongly present among the DEGS (610 in total) of both the H/FH and FH/F contrasts, in both cases among the genes that were down-regulated in the double-stressed berries. In the latter contrast, there were again some enrichments in the up-regulated genes dealing with the response to oxidative stress, also involving genes encoding elements of the “*Glutathione metabolic process*”. Furthermore, a gene coding for a galactinol synthase (*VIT_201S0127G00470*, annotated as *GOLS4*), which was among those validated through qPCR (Figure S2), was upregulated only in the FH berries, similar to what was found by Pillet et al. [25].

After the recovery period, at 9 days after the end of stress imposition (T4), the number of total DEGs continued to show a decreasing tendency in almost all the contrasts, with 21 DEGs in C/F with just two enriched terms, 32 in H/F with no enriched term, 133 in FH/F with seven enriched terms (all up-regulated, most of which dealing with stress, oxidation, and heat), 177 in H/FH with no enrichment, and 340 in C/H. The latter comparison showed three functional enrichments dealing with hormones, i.e., auxin and ethylene, including, in all cases, genes with a down-regulation in the stressed berries with respect to the control ones. At T4, the only contrast with a high number of DEGs (1578) was C versus FH, which opposes the most stress berries to the control ones. Functional enrichments were found only for the down-regulated genes (1189), with terms such as “*Photosynthesis*”, “*Response to oxidative stress*”, “*Oxidation-reduction process*”, “*Protein folding*”, and related annotations. Therefore, although all the other berries had almost completely recovered from stress, the double-stressed ones were still compromised and trying to recover from oxidative stress.

A huge increase in total DEGs occurred at harvest (T5), in correspondence with physiological ripening. Except for C/F and H/FH contrasts, showing 7 and 78 DEGs, respectively, all the other comparisons showed long lists of genes with differential expressions. The biggest amount of DEGs was found for C/FH (3351), followed by FH/F (2795) and C/H (2679). The contrast H/F gave a lower number, i.e., 1112 DEGs (Figure 5). DEGs from C/H showed a significant enrichment by GO terms in nucleic acid and RNA metabolism and gene expression regulation for the 1116 up-regulated genes, similar to the previous contrast. Moreover, the 1563 down-regulated genes were enriched in GO terms dealing with the cell wall and cytoskeleton organization and carbohydrate and polysaccharide metabolism, as well as light harvesting in photosystem I. GO terms related to sterol/steroid biosynthesis were also detected that could be related to the basic components of cellular membranes rather than to a brassinosteroids signaling, since these hormones are usually active at the pre-véraison developmental stage [54]. The GO terms significant enriched in C/FH comparison were related to nucleic acid and RNA metabolism and gene expression regulation for the 1601 up-regulated genes, while the 1750 down-regulated genes were enriched by GO terms associated with peptide and amide biosynthesis/metabolism and protein localization. Comparing the stressed berries, i.e., H and FH, with the ones that suffered flooding in spring (F), a relevant overlapping in terms of GO terms enrichment was observed. Both comparisons (H/F and FH/F), indeed, showed several common terms when analyzing the down-regulated genes (464 and 1431, respectively), such as “*Regulation of gene expression*”, “*Nucleic acid metabolic process*”, “*Regulation of macromolecule biosynthetic process*” and other generic annotations. On the other hand, GO terms enrichment of up-regulated genes (648 and 1364, respectively) differed between the two contrasts. While in H/F, there were enriched terms dealing with “*Cytoskeleton organization*” and “*Photosynthesis, light harvesting in photosystem I*”, in the FH/F comparison, enriched terms dealt with peptide and amide biosynthesis and metabolism, as well as other terms related to cellular macromolecules biosynthesis and protein-membrane insertion. Finally, when comparing the two stressed berries at T5, i.e., H/FH, results showed just three enriched terms for the 66 up-regulated genes: “*Cell wall organization and biogenesis*”, “*External encapsulating structure organization*”, and “*Circumnutation*”.

Based upon the genes previously identified by Dimopoulos et al. [55]. we finally analyzed the amount of transcripts coding for different elements, both enzymatic and regulatory, involved in the cuticular aliphatic wax biosynthetic pathway, trying to further shed light on the aspects discussed above regarding the higher temperatures of both F and FH berries with respect to the C and H ones. Most of the genes did not show any significant variation, except for that putatively encoding the transcription factor DEWAX, found to negatively regulate cuticular wax biosynthesis in Arabidopsis [56] with clear indications of a similar role also in grape [55]. This gene, namely *VIT_216s0013g01000*, was significantly down-regulated from T0 to T1 in both F and FH berries (Figure S3), thus possibly explaining a higher rate of cuticular wax biosynthesis, a lower transpiration rate and, thus, the higher temperatures observed with respect to C and H. Thereafter, the transcript levels in all berries converged to similar values, but the waxes accumulated on the cuticle may have affected their cooling capacity for the rest of the experiment.

### 2.3. Hormones

**Figure 7:**
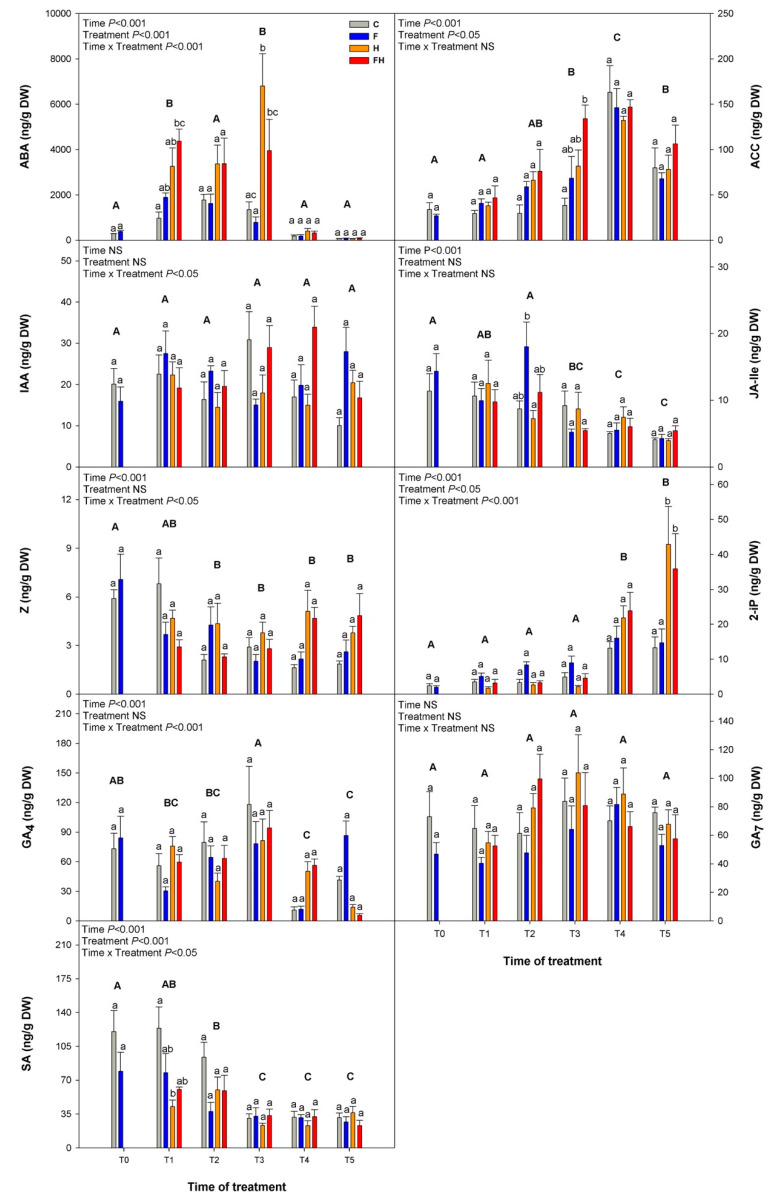
Hormone levels in berries samples (C, control; F, flooded; H, heatwave; FH, flooded plus heatwave). The results of the statistical analyses are reported within each chart along with the letter indicating statistically different values (uppercase for the timepoint and lowercase for the treatments).

Analysis of the levels of key phytohormones in Sauvignon Blanc berry samples showed, as expected, highly significant differences in ABA content between treatments and sampling timepoints (Figure 7; other hormone precursors/metabolites are shown in Figure S4).

As expected, T1, T2, and T3 were the ones presenting the highest ABA content, as its accumulation starts at the onset of the ripening process [57]. At the beginning of the stress (T0; 46 DAA), minimum levels of ABA were detected, resulting in no significant differences between the C and F berries. At T1, H and FH berries accumulated more ABA than C and F, showing significant differences between treatments, especially comparing C berries to FH, which might eventually cause an advance in the ripening process [14]. While at T2, the situation was stable, at T3, the H berries showed significantly higher levels (about five-fold) when compared to the C and F plants. The 9-cis-epoxycarotenoid dioxygenase (NCED) is a well-known step-limiting enzyme in the biosynthesis of ABA [58], and studies in other fruits such as oranges or avocados found that this enzyme can be induced by drought stress [59,60], most likely causing a *de novo* synthesis of ABA [58]. In our experiment, differences in the expression levels of the gene *VIT_219s0093g00550* that encodes for NCED3 were found at the beginning of the stress period, possibly justifying the increased levels of ABA in H and FH berries at T1 (Figure S5). The same gene was also strongly up-regulated in H berries at T3, consistent with the ABA peak observed at this timepoint when both stresses, i.e., drought and heat, were at maximum levels. In addition, *VIT_202s0087g00930* (*NCED4*), *VIT_210s0003g03750* (*NCED5*), and *VIT_205s0051g00670* (*NCED6*) genes showed increasing expression levels in stressed berries, but only at T2 and T3, likely supporting the enhanced amount of ABA observed during this late phase. Although *NCED3* has a pivotal role during stress-induced ABA accumulation in different tissues [61], it is known that also *NCED5* can be induced during drought/heat stress in a coordinated manner with *NCED3* [62], which is consistent with our observations. Studies with ABA-insensitive mutants showed a strong susceptibility to water deficit and heat stress, not being able to accumulate important proteins such as Heat Shock Proteins (HSP), multiprotein bridging factor 1c (MBF1c), or ascorbate peroxidase 1 (APX1), as many of these transcripts are ABA-responsive [63]. *VIT_217s0000g07190*, which codes for HSP101, presented high expression levels in berries undergoing heat stress (H) and combined stress (FH) from T2 until the recovery in T4, indicating that this gene might be ABA-dependent. After 9 days of recovery (T4), results showed a drastic decrease in ABA contents in all the berries, which continued to decline until the end of the experiment, when berries were harvested at 74 DAA, to levels even below the ones found at the beginning of the experiment. This decline in the ABA levels during the last stages of berries ripening might be catalyzed by ABA-8′-hydroxylase, a cytochrome P450 monooxygenase (CYP707A), which is responsible for the rapid inactivation of ABA in plants [58]. Among the seven genes annotated in grapevine as encoding CYP707A enzymes, only one seemed to play a role in ABA decline from T3 to T4, that is *VIT_218s0076g00340* coding for CYP707A3, which showed a strong increase in its expression with a peak at T3, right before the drop of ABA levels (Figure S5). ABA contents can change drastically as a response to dehydration and rehydration conditions by the induction of CYP707A1 and CYP707A2 in Arabidopsis [64]. Under our conditions, however, none of these gene homologs was induced, and the up-regulation of *CYP707A3* in all the berries suggests that the control of ABA catabolism is prevalently under developmental cues and not affected by rehydration.

The importance of ethylene signaling during the ripening of grape berries has been previously described, despite the technical difficulties in measuring its biosynthesis in a reliable way in this crop [65,66]. Being 1-aminocyclopropane-carboxylic acid (ACC), the limiting-step direct precursor of ethylene, its content was analyzed, showing a progressive increment from the beginning of the experiment, with statistically significant differences between timepoints, and reaching its maximum at T4 after the recovery period, followed by a slight decrease at harvest time in all four treatments (Figure 7). Significant differences between treatments were only detected at the end of the heatwave (T3, at maximum stress), specifically between C berries, with ACC contents below 50 ng/g DW, and FH berries, with concentrations of ACC about three-fold higher than the C berries. Within this context, ACC biosynthesis might be regulated by ACC synthase 1 (VvACS1) since the gene *VIT_215s0046g02220* coding for this enzyme showed a consistent differential expression between timepoints. ACS1 is a type I ACS that may be activated under several stresses, leading to enhanced ethylene production, as previously reported [67,68,69]. ACC transformation into ethylene is controlled by ACC oxidases, encoded in grapevine by three genes, named *VvACO1* (*VIT_211s0016g02380*), *VvACO2* (*VIT_212s0059g01380*) and *VvACO3* (*VIT_00s2086g00010*). Previous studies pointed out the different roles of these genes in berry ripening [70], with *VvACO1* and *VvACO2* playing a pivotal role. While the former showed a generally increasing expression with higher values at T1 for both F and FH berries, consistent with the advanced ripening observed in these berries, the latter was more correlated with *VvACS1* and, thus, with the stress response, displaying a consistent peak of expression at T2 (Figure S5). These results suggest a positive involvement of ACC and ethylene in the ripening process of Sauvignon Blanc grape berries in accordance with other studies in grapevines [71] and, on the other hand, an accumulation of ACC as a response to extreme stress conditions.

Results regarding auxin (Figure 7) showed a generally low accumulation of indoleacetic acid (IAA) in berries, and these levels did not show any significant difference between timepoints, or stress treatments applied to the grapevines.

In the case of the conjugate jasmonoyl-isoleucine (JA-Ile; Figure 7), the endogenous bioactive form of jasmonate [72], a continuous decrease in its levels during the ripening of berries until T5 was observed. Only at T2, after four days of the heatwave, berries that underwent a spring flooding (F) presented a statistically significant higher accumulation of JA-Ile compared to the other treatments. Similar to IAA, endogenous levels of JA-Ile are high during the first developmental stages of grape berry fruit to promote cell division and fruit set [73]. Moreover, JA-Ile participates in environmental responses in plants [74,75], though this is not supported by our data.

The two bioactive cytokinins (CKs) zeatin (Z) and 2-isopentenyladenine (2-IP) showed diverging patterns of accumulation in Sauvignon Blanc berries (Figure 7), with a declining tendency throughout the experiment for the former and an increasing trend for the latter. CKs are important plant growth regulators, as they actively participate during fruit set and early cell division, generally decreasing thereafter during the ripening phase in most fruits [76]. However, a few recent studies interestingly showed that depending on the fruit and also on the specific cultivars, CKs may increase during ripening. This was observed in kiwifruit for Z [77] and in the grape berry for 2-IP [78], in the latter case, with a good correlation of several CK-related genes encoding both biosynthetic and regulatory proteins [73]. The expression profiles of most of these genes largely overlap with the patterns of transcription observed in our unstressed samples, and some of them show a possible involvement in the stress response (Figure S6). In the same study, Sauvignon Blanc was shown to be a moderate accumulator of 2-IP after véraison when SSC was in the range 19.4–20.8 °Brix, which corresponds approximately to the T4 sample of the present trial. Indeed, the levels of 2-IP in the C and F unstressed berries reached their maximum at T4, although the stressed samples displayed higher levels at T5 (statistically significant, *p* < 0.05). Several studies demonstrated an active role for CKs in abiotic stress tolerance [79,80], as an inductor of osmoprotectants, such as proline, in drought responses [81]. Since proline is one of the amino acids mostly induced in stressed samples (see next section on NMR), this marked increase in 2-IP in fully ripe berries may indicate a specific role for this bioactive cytokinin in both stress tolerance and the long-term recovery from stress.

As far as the gibberellins (GAs) are concerned, only GA_4_ and GA_7_ were detected in the berry samples. The statistical analyses did not point out any significant difference consistent with the experimental treatments for either of the GAs, although some differences between the timepoints were displayed by GA_4_. However, if we look at the general trends, some interesting observations can be made. For example, at T4, after the recovery period, GA_4_ content is about three-fold in the stressed berries with respect to the unstressed, while GA_7_ showed a slightly increasing trend in stressed samples, peaking at T3 and T2 in H and FH berries, respectively. These trends are also supported by the expression patterns of some GA biosynthetic/metabolic genes (Figure S7), among which three GA3-oxidase (GA3ox), six GA20-oxidase (GA20ox), and nine GA2-oxidase (GA2ox) genes that were identified based upon similarity versus the *Arabidopsis thaliana* genome. In detail, the expression of *VIT_209s0002g05270*, coding for a GA3ox, peaked at T1 and T2 in H and FH berries, although its general levels of expression are low. On the other hand, *VIT_215s0048g01320*, encoding a GA20ox, showed higher expression levels and peaked at T2 and T3 more consistently with GA_7_ content. The *GA2ox* gene *VIT_210s0003g03490* followed a similar pattern, supporting the hypothesis that the last two transcripts may be involved in GA_7_ biosynthesis and specific inactivation in the berry as a response to stress. Indeed, it was previously shown that *GA2ox* genes are induced by gibberellins themselves as a homeostatic mechanism [82]. The last *GA2ox* gene, *VIT_210s0116g00410*, showed expression patterns paralleling those of GA_4_ at T4, supporting the idea that, after the recovery period, the stressed berries were still trying to inactivate gibberellins, which are considered ripening inhibitors [83].

The amount of salicylic acid (SA) showed a statistically significant decreasing trend until T3, with higher values observed in the unstressed samples, especially the C berries, from the beginning of the experiment up to T2 (statistical significance only at T1). At T3, SA content decreased in all treatments and remained at lower levels until the end of the experiment (Figure 7). SA is known for improving fruit quality when applied exogenously at pre-vèraison by enhancing anthocyanins contents [84], as well as improving and extending the berry’s shelf life [85]. However, SA also acts as a ripening inhibitor [86], so its decrease is consistent with previous findings. In parallel, the gene *VIT_217s0000g05750*, coding for isochorismate synthase (ICS) that converts chorismate into the immediate SA precursor isochorismate, showed decreasing expression levels in all four treatments (Figure S7). Although SA was previously shown to be linked to several physiological responses to biotic and abiotic stress [87,88,89], including heat stress responses in plants, by reducing the oxidative damage when applied as a pre-treatment [90], our results showed no evidence of SA involvement in berries undergoing stress, with the only exception of a statistically significant decrease at T1 in H berries.

**Figure 8:**
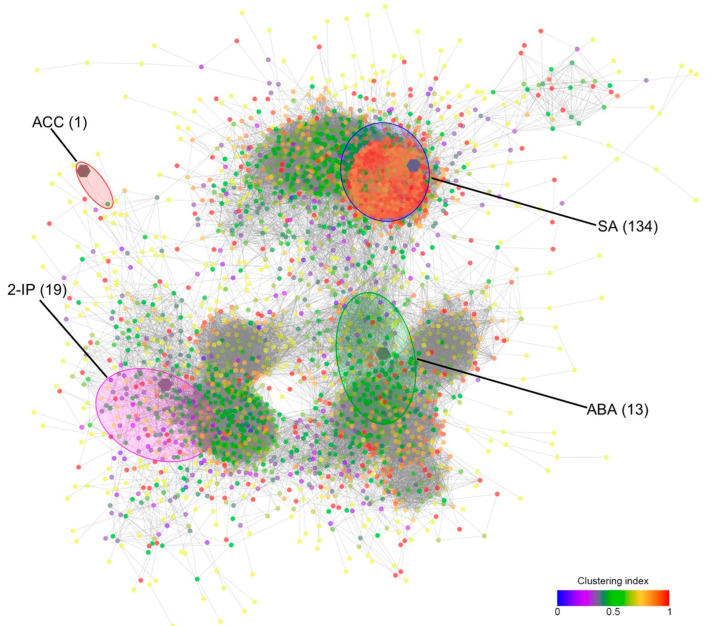
Correlative network built with genes (circles) and hormones (hexagons) according to their correlation index (ABA, abscisic acid; ACC, 1-aminocyclopropane-carboxylic acid; 2-IP, 2-isopentenyladenine; SA, salicylic acid). Only nodes with correlations higher than 0.8 in their absolute values were included. Node color represents the clustering index as shown in the bottom-right color scale. Subnetworks, including the first neighbor nodes directly connected to each hormone, are also shown with ellipses of different colors and the number of genes between parentheses. The network is shown with the ‘Prefuse Force Directed’ layout based on the parameter ‘Edge Betweenness’.

As a complementary approach, in order to ascribe a functional meaning to the different hormones showing significantly higher or lower levels with respect to their control samples as related to the different stress conditions and combinations, a correlation network approach was attempted using both the hormones quantification data of ABA, ACC, 2-IP and SA, and the expression levels of a selected subset of genes (coefficient of variation ≥ 1; the sum of normalized expression ≥ 1; |correlation coefficient| ≥ 0.8). Following the assumption that a strong correlation between a hormone and some genes may indicate a functional relationship, the nodes connected to each hormone inside the network may provide useful indications. The correlative network consisted of 4773 nodes connected by 1,455,742 edges (Figure 8). Surprisingly, the most relevant subnetwork included SA and 134 genes (i.e., 134 neighbor nodes directly connected to SA) with the highest mean clustering index (0.78), followed by the more dispersed subnetworks of 2-IP (19 genes), ABA (13 genes), and ACC with just one gene.

The functional enrichment of the SA network pointed out GO terms dealing with “*Transcription factor activity*”, “*Response to endogenous stimulus*”, and “*Signal transduction*” (Table S3). Among the 38 non-redundant genes annotated with such enriched GO terms, seventeen encoded transcription factors, such as ERF/EREBF, MYB, NAC, bHLH, WRKY, LOB, and Homeodomain, most of which are known to be involved in the ABA-independent responses to drought and associated stresses [91]. However, as pointed out above, the levels of SA did not show any specific relationship with ongoing stress, and in most cases, particularly at T1 (statistically significant) and T2 (non-significant), its amount in stressed berries was even lower than in the unstressed ones (Figure 7). Among the most specific GO terms enriched in genes belonging to SA subnetwork there is also “*Ripening*”, with two genes associated with it, coding for a pectin lyase (*VIT_207S0005G02090*) and an ACC synthase (*VIT_202S0025G00360*), both previously shown to be induced at ripening inception to decrease thereafter [53,92] as confirmed by their expression patterns (Figure S7).

As far as the 2-IP subnetwork, despite no enriched GO term, there are some relevant genes correlated with the levels of this interesting hormone that can help to shed light on the function of this cytokinin during the late ripening stages of the grape berry, close to senescence, especially in the stressed fruits. First of all, there are two transcription factor genes, *VIT_208s0007g04180* and *VIT_207s0031g02280*, indicating that the berry was still transcriptionally active at T5 when their expression peaked together with the transcripts levels of a gene encoding a senescence-associated protein (*VIT_201s0146g00260*) correlated with 2-IP levels as well. Moreover, one gene coding for a ribosomal protein (*VIT_200s0873g00010*), two coding for wound-responsive proteins (*VIT_212s0057g00110* and *VIT_212s0057g00080*), and a last one coding for a terpene cyclase (*VIT_209s0054g01410*) were highly expressed in both H and FH berries, with basal levels in unstressed berries. This would indicate that 2-IP may not only play a role in late-ripening/senescence but also coordinate a long-term response to stress with active protein synthesis, a response to wounding, and the activation of terpenoid metabolism, the latter possibly resulting in a specific production of aromas in stressed berries.

Although the thirteen genes of the ABA subnetwork did not show any significant functional enrichment taken as a whole, most likely because of the scarce numerosity, the analysis of every single gene may provide key information about the role of ABA from T1 to T3 in the stressed berries. Apart from two transcripts with putative unknown functions, it is worth noting that three of these genes code for transcription factors, i.e., two MYBs (*VIT_207s0005g01950* and *VIT_219s0015g01280*) and a MADS-box (*VIT_210s0042g00820*), known to be involved in response to stress in different species and crops [93,94]. In particular, the putative ortholog of the gene *VIT_207s0005g01950* (annotated as *MYB108*) was shown to confer drought tolerance in herbaceous peony [95] and regulate the responses to ROS (Reactive Oxygen Species)-mediated signaling from both abiotic and biotic stresses in Arabidopsis [96]. It is worth noting that among all the ABA-correlated genes, the *MADS-box* transcript showed the earliest induction, with a visible increase in its expression in stressed berries already at T1 (Figure S8). Seven genes encoded enzymatic proteins with different putative activities, such as a DNAse I-like superfamily protein (*VIT_201s0011g06310*), a matrix metalloproteinase (*VIT_201s0026g00900*), a CYP704B1 (*VIT_201s0026g02700*), a Pyridoxal phosphate (PLP) phosphatase-related protein (*VIT_204s0008g00870*), a Histidine Kinase (*VIT_204s0069g00750*), a Lactoylglutathione lyase/glyoxalase I family protein (*VIT_209s0002g06430*), and a Thioredoxin superfamily protein (*VIT_210s0003g00390*). Matrix metalloproteinases (MMPs) are involved in the remodeling the plant’s extracellular matrix during growth, development, and stress response [97] in different organs, such as the root [98] or other vegetative or reproductive parts of the plant. In rice, a matrix metalloproteinase plays an important role in symplastic-apoplastic transport by modulating cellulose and callose depositions [99], a potentially important function considering that grape berries undergo a shift of phloem unloading from symplastic to the apoplastic pathway at the onset of ripening [100]. Members of the Cytochrome-P450 gene family are known to be involved in core metabolisms, and the clan CYP86, to which CYP704B1 belongs, was shown to be responsible for cutin and suberin biosynthesis and thus control water evaporation and transpiration in several species [101,102,103]. Moreover, in Arabidopsis, these enzymes were shown to reduce cuticle membrane thickness, thus increasing the tolerance of plants against drought by increasing water permeability [104]. Histidine kinases may control abiotic stress signaling, although a negative role in drought stress was proposed in Arabidopsis for AHK5 [105], along with an ABA-independent function in integrating multiple signals via H_2_O_2_ homeostasis in guard cells to control stomata closure [106]. B_6_ vitamers (VB_6_), in addition to being important enzyme cofactors in several biochemical transformations, were shown to have additional functions as ROS scavengers [107], resistance factors to biotic and abiotic stress in plants [107], and many other roles [108]. Within this context, the PLP-phosphatase gene may play a role in VB_6_ metabolism of stressed berries, as observed in tobacco plants under different abiotic stress conditions [109]. The other two ABA-correlated enzyme-coding genes may be involved in specific detoxification mechanisms (glyoxalase; *VIT_209s0002g06430*), and regulation of the redox environment within the berry (thioredoxin; *VIT_210s0003g00390*), which was shown to be significantly compromised based upon visual analysis of stressed bunches (Figure S9). The glyoxalase pathway is required for detoxification of cytotoxic metabolites that would otherwise be lethal. Glyoxalases are known to be differentially regulated under stress conditions and their overexpression in plants confers tolerance to multiple abiotic stresses [110] among which drought [111,112,113]. It was also shown that glyoxalases also play a role in maintaining GSH homeostasis and subsequent ROS detoxification [114], therefore these enzymes may collaborate also with thioredoxin, whose gene correlated with ABA in stressed berries as well.

### 2.4. NMR Metabolic Profiling

**Figure 9:**
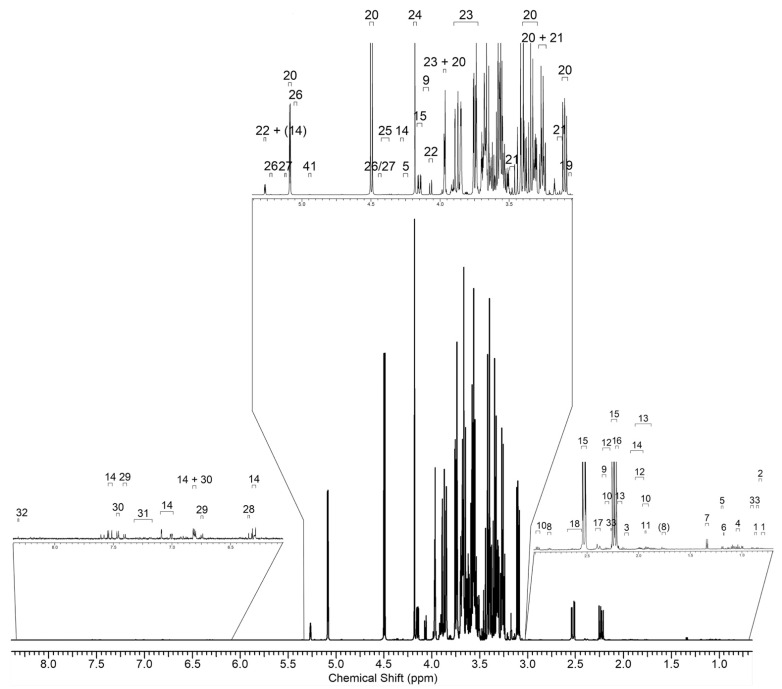
Representative ^1^H-nuclear magnetic resonance (NMR) spectrum of *Vitis vinifera* berry extract in D_2_O phosphate buffer, pH 6.86. Three portions of the spectrum were magnified to show resonances that have been assigned and labeled (1, isoleucine; 2, leucine; 3, valine; 4, ethanol; 5, threonine; 6, lactic acid; 7, alanine; 8, lysine; 9 proline; 10, GABA; 11, acetic acid; 12, glutamine; 13, glutamic acid; 14, chlorogenic acid; 15, malic acid; 16, acetoacetic acid; 17, citric acid; 18, aspartic acid; 19, malonic acid; 20, glucose; 21, myo-inositol; 22, sucrose; 23, fructose; 24, tartaric acid; 25, DHA; 26, xylose; 27, galactose; 28, fumaric acid; 29, vanillic acid; 30, tyrosine; 31, phenylalanine; 32, formic acid).

A metabolomic fingerprint was carried out through NMR on berry samples collected at T2 and T4. The choice of T2 samples was based upon the transcriptomic analyses, which pointed out the highest number of DEGs in the stressed berries at this timepoint. T4 was considered because, at this phase of the experiment, a normal physiological status was supposed to be recovered, as substantially confirmed by gene expression analysis. In addition to providing a fingerprint-based on both polar and hydrophobic metabolites, NMR also pointed out a relative quantification of known metabolites, identified through several complementary methods as described in the Materials and Methods Section 3. An example of the NMR spectrum is shown in Figure 9, along with peak identification.

**Figure 10:**
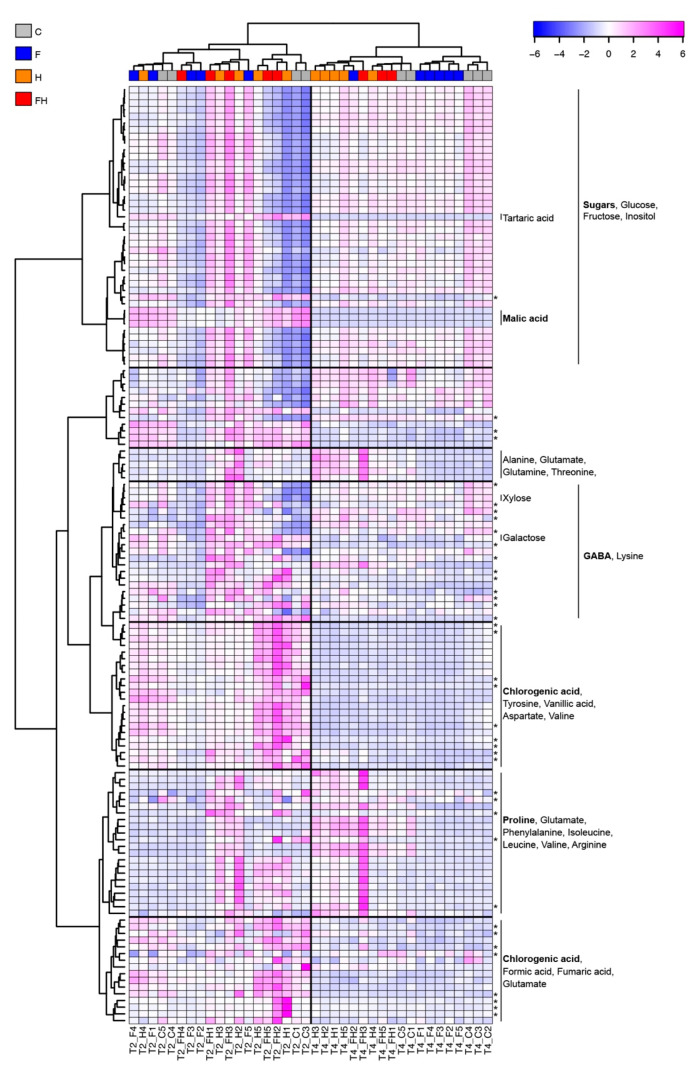
Heatmap of the signal area values of compounds quantified through NMR in T2 and T4 samples. The different replicates were marked at the top of the heatmap with different colors according to the sample to which they belong (a legend is provided at the top left), while the scale of the heatmap values is shown at the top right. Samples are named according to the timepoint, the treatment (C, control; F, flooded; H, heatwave; FH, flooded plus heatwave), and the replicate. (*, Unknown compound).

Signal area values were used to build a heatmap summarizing all the results (Figure 10), except for two outliers showing very anomalous metabolic profiles (T2_C2 and T2_FH4), which were discarded from this analysis. This is quite a common problem with grape berries, especially when samples are collected during ripening progression, which is not homogeneous along the bunch [115,116]. After removing this small bias, T2 berries were very well separated from those of T4, as shown by the hierarchical clustering applied to samples. This first observation may support the reliability of this analysis, which is not always obvious with metabolomic profiling on this type of fruit, at least in discriminating the two timepoints.

Regarding the separation of stressed from unstressed samples, the situation is slightly different. At T2, there was no clear separation between the treatments, although two metabolic markers of ripening progression belonging to the same group of the sugar compounds, such as tartaric and malic acids, showed values that were consistent with both development and the stress that occurred. Considering that both acids are known to decrease after véraison [117] and taking into account their variations also at T4, our data fully match the known trends (Figure 10). Moreover, lower values with respect to the control were observed for both acids in the F berries, most likely due to their advanced ripening, and also in the heat wave-stressed samples, consistent with previous studies claiming the negative effects of high temperatures and global warming on berry acidity [23,118,119,120]. Concerning the other sugar compounds, all grouping together in the heatmap, the situation reflected quite well the SSC shown above in Figure 2, especially at T4, when the C and F berries showed the highest values.

Differently from previous studies pointing out an increase in sugars, such as glucose, fructose, and sucrose, during drought stress (for a review, see [121]), a detailed view of these single compounds quantified through NMR did not point out any consistent trend (Figure S10 and data not shown). Concerning the other compounds detected through NMR, two main classes of metabolites were markedly shown to increase upon stress, i.e., the amino acids and chlorogenic acid (CGA). Concerning the former, we can further subdivide the amino acids into those produced by stressed berries mainly at T2, such as tyrosine, those mostly found at T4, such as proline, and those detected at both timepoints, such as alanine and γ-aminobutyric acid (GABA), while CGA was prevalently produced upon stress at T2 (Figure 10). A relevant amount of the literature reports several findings about the involvement of these compounds in response to different types of stress, although the reaction is often species-specific. For example, tyrosine was previously found to be involved in drought stress response in Arabidopsis [122,123,124,125], *Haberlea rhodopensis* [126], wheat [127], maize [128,129], as well as in heat stress reaction in maize [130] and Arabidopsis [125].

For proline, previous studies indicate not only its involvement in drought stress as an osmoprotectant [122,126,127,131,132,133,134,135,136] but also an intriguing specific role in tuning the redox potential conferring tolerance to high temperatures [137] as previously demonstrated [125,138]. Moreover, proline production can be induced by CKs, as previously demonstrated [81], and since 2-IP was shown to increase starting from T4, especially in the stressed berries (Figure 7), a causal relationship may be hypothesized between the increased levels of CK and proline.

In addition to the little evidence regarding the involvement of alanine in heat stress [125], several studies pointed out the relevant role of GABA. Another amino acid was found to increase in stressed berries. GABA-mediated improvement of plant tolerance to drought was previously shown in Arabidopsis [122,125], rice [136], soybean [134], tomato [131], and maize [128]. GABA may act through both antioxidant activity and cooperation with other molecules, and its key role in drought stress resistance in plants is a matter of debate [139]. For example, proline, alanine, and glutamate are all metabolites associated with GABA accumulation in plants in response to ROS generation [140,141,142] and were all found to increase in stressed berries of our experiment. In general, although some amino acids may have specific roles, as reported for proline [137], the general increase observed for these primary metabolites in stressed berries may be caused by the degradation of highly abundant proteins, which generates an accumulation of free amino acids that are catabolized as an alternative respiratory substrate to compensate for the decreased photosynthesis caused by drought and heat [143]. Regarding CGA, a phenylpropanoid whose abundance at T2 in stressed berries was clearly shown through NMR (Figure 10 and Figure S11), a role as an antioxidant can be hypothesized as previously shown in oat [144,145] and tobacco [146], and specifically during drought stress in Arabidopsis [147] and poplar [148].

Consistent results were found from expression patterns of some specific genes involved in the biosynthesis and metabolism of the above compounds (Figure S12). Concerning the accumulation of amino acids, such as proline and GABA, since nitrogen assimilation is limited during drought stress, an alternative source of ammonium may derive from glutamate, which is synthesized by glutamate dehydrogenase (GDH) that catalyzes the formation of this amino acid by the amination of 2-oxoglutarate coming from the TCA cycle [149]. Among the seven grapevine genes encoding GDH, two (*VIT_216s0039g02750* and *VIT_216s0039g02720*) were found to be strongly up-regulated in stressed berries. In addition, proline biosynthesis generally occurs as a result of ∆^1^-pyrroline-5- carboxylate synthase (P5CS) and ∆^1^-pyrroline-5-carboxylate reductase (P5CR) catalytic activities in cytoplasm or chloroplasts. *VIT_213s0019g02360* (among the three *P5CS* genes) and *VIT_208s0007g05600* (the only *P5CR* gene) were shown to be overexpressed from T3. We also investigated the expression patterns of key genes involved in phenylpropanoid biosynthesis and found that two genes coding for hydroxycinnamoyl-CoA shikimate/quinate hydroxycinnamoyl transferase (HCT), a key enzyme in CGA biosynthesis, were up-regulated in stressed berries from T2 (*VIT_209s0018g01190*) and T3 (*VIT_211s0037g00440*).

### 2.5. Modelling the Berry Response to Stress

**Figure 11:**
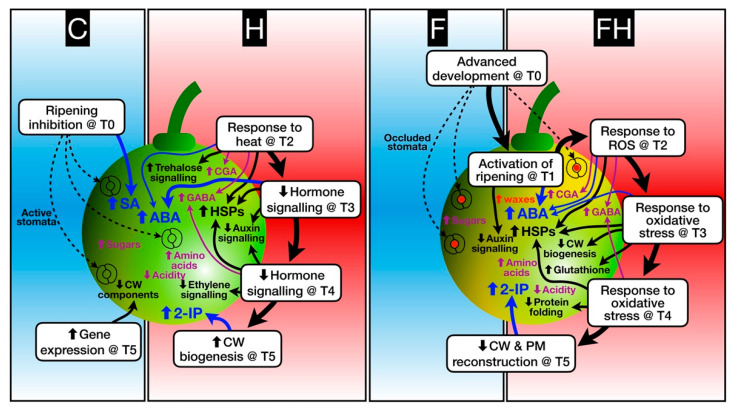
Model summarizing the specific responses of the berry to each treatment (C, control; F, flooded; H, heatwave; FH, flooded plus heatwave). The position of each item with respect to the background boxes, both outside and inside the stylized berries, refers to the specificity of the response. When an item overlaps both boxes, it means that the particular response was shown in both treatments. The main transcriptional responses inferred from RNAseq data and GO terms enrichment analyses are shown with black text on the white background outside the berry. Black arrows link these general responses to more detailed transcriptional information (inside the berry), while the blue arrows connect with hormonal responses inferred from hormone profiling. Finally, the metabolic responses are shown in violet and can be either linked to a particular timepoint response outside the berry or not. In the latter case, the metabolic response was generally observed during the whole experiment. Stomata are also shown, either active or occluded, by waxes (red circle), as discussed in the main text. (2-IP, 2-isopentenyladenine; ABA, abscisic acid; CGA, chlorogenic acid; CW, cell wall; GABA, γ-aminobutyric acid; HSPs, Heat Shock Proteins; PM, plasma membrane; ROS, reactive oxygen species; SA, salicylic acid).

In order to summarize the most relevant results and achieve an overview of the berry response to every single experimental treatment, we built a model (Figure 11). According to this model, at T0, both F and FH berries show an advanced development with respect to other treatments (C and H). We can speculate that the flooding that occurred at bud-break may have affected flower development determining a sort of ‘memory-effect’ inducing the berry to develop and ripen faster. Future investigations may shed light on this aspect, eventually taking into account also the epigenetic modifications that can work as a medium-term memory in plants [150]. Such a ripening advance most likely caused an increased synthesis of epicuticular waxes, which contributed to stomata occlusion and a consequent reduction in transpiration rate, thus causing F and FH berries to warm up faster than C and H. The trends in SA content confirm that in C berries, ripening was still inhibited when the trial began. Indeed, at T1, F and FH berries already displayed the specific activation of the ripening program, as testified, among the other transcriptional markers, by auxin signaling genes, which were down-regulated, and by the increase in ABA, which was even at higher levels in the stressed berries. However, while in the FH fruits, ABA peaked at T1, in the H berries, it peaked later at T3. Differential response to the heatwave was observed between FH and H berries. Indeed, the former berries responded mainly to ROS and to general oxidative stress from T2 to T4. This reaction was closely linked to an up-regulation of HSP-genes from T2 to T3, followed by a down-regulation of cell wall biogenesis and an up-regulation of glutathione metabolism at T3, and continuing again at T4 with the down-regulation of protein folding. The response of FH berries to the heatwave was accompanied by an increased amount of CGA, GABA, and other amino acids, such as tyrosine, proline, and alanine, all of which are potentially involved in response to heat stress. The situation of FH berries was critical until T4 and started to recover only at T5, with the activation of the cell wall and membrane reconstruction. The H berries responded to the heatwave in a more conservative way by showing a ‘classical’ heat response at T2 involving trehalose signaling and the induction of HSP. At the same time, the levels of CGA and GABA increased such as in the FH. As said before, at T3, the maximum ABA levels were reached, in parallel with the down-regulation of auxin signaling typical of ripening inception, which continued at T4, also affecting some ethylene-related genes, indicating the full recovery of normal berry development. At T5, cell wall biogenesis was active, indicating a progression of the ripening syndrome similar to that occurring at T5 in the reference C berries. Despite these differences, both H and FH berries showed high levels of 2-IP at T5, which may guarantee a long-term recovery from stress as an inductor of osmoprotectants, such as proline, in both situations.

## 3. Materials and Methods

### 3.1. Plant Material, Experimental Setup, and Sampling

**Figure 12:**
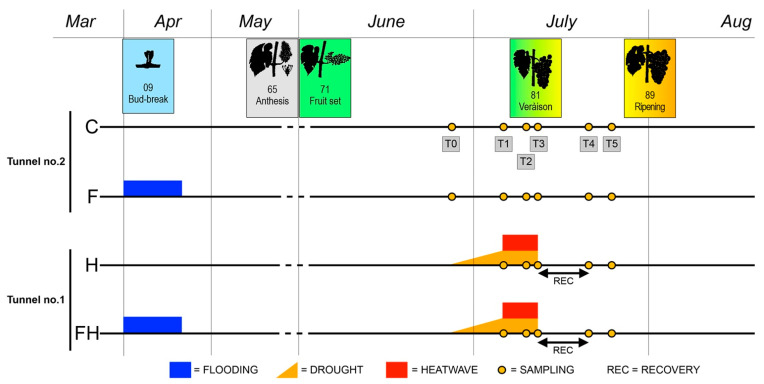
Schematic representation of the experimental set-up. The months of the year are reported at the top, with the key phenological stages indicated below (BBCH scale; [44]). The four experimental treatments (C, control; F, spring flooding; H, summer heatwave; FH, spring flooding + summer heatwave) are shown as separate lines, along with the key time points for sample collection, with the indication of the tunnel to which they belong on the left. The legend on the bottom describes the colors used in the scheme.

Experiments were carried out in 2017 and 2018 on potted grapevines, *Vitis vinifera* cv. Sauvignon Blanc (clone 108) grafted onto Kober 5 BB (K5BB) (*V. berlandieri* × *V. riparia*) rootstock. A suitable number (400) of three-years-old plants were grown in 10L pots filled with a sand–pumice-peat mixture (1:1:3 in volume) under partially controlled conditions in two plastic film tunnels fully with continuous ventilation (to avoid overheating) and optimal irrigation located at the Experimental Farm of the University of Padova “L. Toniolo” in Legnaro, north-east of Italy. Plants were randomized into four groups, according to homogeneous developmental characteristics (i.e., length of the cane, caliper, and a number of buds) and pruned before budburst with three or four dormant buds per plant retained. Half of the plants for each tunnel underwent waterlogging stress during spring, approximately at budbreak (BBCH stage 09), as previously described [151]. At the 5-separated-leaves stage (BBCH stage 15), the plants were thinned to two shoots and trained vertically. Just before véraison, at 46 DAA (BBCH stage 79–81) [44], a heatwave was imposed in Tunnel no.1 by simulating the natural time course of such extreme events, i.e., an initial phase of drought and, when soil water content reached 30% of field capacity (after 9 days), a second phase during which temperatures were increased by setting up the thermostat at 5 °C higher than Tunnel no.2 for six consecutive days (Figure 12). Sample pots were selected during soil preparation and were weighed before and after saturating the soil with water at 100% field capacity (Fc 1.0) to calculate the soil water content (g of water/g of dry soil in the pot) at Fc 1.0. The water supply was progressively reduced to 30% of Fc 1.0, and water stress conditions were assessed when the average reading of the stress-exposed pots reached the required soil water content. In order to mitigate the fluctuations in soil water content, all pots were weighed at 6 pm daily and the amount of water needed to maintain the desired soil field capacity was then added.

Samples were collected from all four groups of plants (C, control; F, flooded; H, heatwave; FH, flooded plus heatwave) at the beginning of the stress (T0; 46 DAA), when the soil water content was at 30 % of Fc in stressed plants (T1; 55 DAA), at four days after temperature increase (T2; 59 DAA), at the end of the heatwave (T3; 61 DAA), after 9 days of recovery (T4; 70 DAA) and, finally, at the ripening of the control grapes (T5; 74 DAA). At each sampling time point, berries were collected from five randomly chosen grapevines per treatment, frozen in liquid nitrogen, and stored separately at −80 °C as five biological replicates for the following analyses.

### 3.2. Meteorological and Biometrical Measurements

Outside and inside each tunnel, meteorological sensors were deployed for the measurement of basic meteorological parameters. Air temperature (°C) and relative humidity (%) (Vaisala HMP45, Helsinki, Finland) and global radiation (W m^−2^) (LI-200R, Licor) were measured every second and averaging every 30 min by a datalogger (CR3000, Campbell Sci., Logan, UT, USA). During heatwave simulation, berry temperature was also measured using 0.076 mm diameter type ‘E’ thermocouples (OMEGA Engineering, Stamford, CT, USA). The thermocouples were inserted into the center of five berries per treatment in exposed clusters.

At each sampling date, 5 clusters per treatment were immediately brought to the laboratory, and berry diameter (*n* ≥ 10), soluble solids content (°Brix), and titratable acidity (% of tartaric acid) were measured using a digital handheld temperature compensating refractometer-acidity meter (mod. PAL-BX|ACID, Atago Co., Tokyo, Japan) on at least five replicates.

### 3.3. RNA Isolation, RNAseq Analyses, and qPCR

Total RNA was extracted using the method of [152] and used for RNAseq at the CRIBI center of the University of Padova using an Illumina NextSeq 500 platform (Illumina, San Diego, CA, United States) allowing to obtain at least ∼24 million stranded single-end reads of 75 base pairs per sample. The whole procedure was carried out following the manufacturer’s instructions. Raw sequence data files were uploaded to the Gene Expression Omnibus (GEO) database (https://www.ncbi.nlm.nih.gov/geo/ accessed on 15 December 2022) under the accession number GSE206753. RNA-seq reads were quality-checked using FastQC (Brabaham Bioinformatics, Cambridge, United Kingdom) and resulted in average, very high quality. Reads were not trimmed before mapping, as the adapter content was negligible. Sequence duplication was moderate, and to avoid problems due to the removal of duplicates in single-end [153], the entire set was mapped. Spliced alignments were performed with TopHat [154], and only uniquely mapping reads (identified by the flag NH:i:1 in the bam alignment file) were used for transcript abundance quantification. Quantification was performed by using the Bedtools coverage program [155]. A revised version of the Grape genome based on the 12X Genoscope Pinot Noir genome [156] and the annotation revised by Vitulo et al. [157] were used for reads mapping, along with the related gff file and GO functional annotation.

cDNA was synthesized with the SuperScript^®^ VILO^TM^ cDNA Synthesis Kit (Life Technologies, Carlsbad, CA, USA) from 300 ng of DNA-free total RNA in a final volume of 20 μL, according to the manufacturer’s instruction. RT-qPCR was performed using StepOne Real-Time PCR System (Applied Biosystems, Monza, Italy) as described by Nonis et al. [158] and Eccher et al. [159], using the PowerUp^TM^ SYBR^TM^ Green Master Mix kit (Applied Biosystems, Monza, Italy). Analyses were performed on three independent biological replicates and three technical replicates for each treatment by using specific primers listed in Table S4. Gene expression levels were calculated using the automated Excel spreadsheet Q-Gene designed by Simon [160], using the modification of the ΔC_t_ method suggested by Pfaffl [161]. Gene expression values were normalized with five *V. vinifera* genes chosen because of their stability of expression as checked by means of the BestKeeper spreadsheet v1.0 [162] starting from the whole set of RNASeq data. Housekeeping primers are listed in Table S4. Expression levels were then reported as an arbitrary unit of Mean Normalized Expression, calculated using equation 2 of the Q-Gene spreadsheet.

### 3.4. Bioinformatic Analyses

The RNAseq counts were analyzed with the online bioinformatic pipeline iDEP version 0.96 [52]. In detail, the raw counts were uploaded to obtain a heatmap and the related hierarchical clustering of the 5000 most variable genes, with distance based on correlation, average linkage, and a cutoff Z score equal to 4. The Principal Component Analysis (PCA), was obtained from the same genes. The Differentially Expressed Genes (DEGs) were identified using DESeq2 [163] with an FDR < 0.05 and a minimum fold change of 1.5. Enrichment analysis was performed for all analyzed contrasts using the DEG2 section with the implemented Gene Ontology annotation. The dot plot of the enriched GO terms was obtained with the software R version 4.1.2 (https://www.r-project.org accessed on 15 December 2022) with the packages dplyr, ggplot2, and forcats.

The network analysis of hormones and gene expression levels was performed with the package Expression Correlation in Cytoscape version 3.9.1 [164]. In this analysis, only the genes with a coefficient of variation ≥ 1, a sum of normalized expression ≥1, and an absolute value of correlation coefficient ≥ 0.8 were included, along with the hormones showing at least a statistically significant difference in the time course. The network was visualized with the ‘Prefuse Force Directed’ layout based on the parameter ‘Edge Betweenness’ calculated through the Network Analysis tool of Cytoscape, and nodes were colored according to their clustering index calculated in the same way.

The hierarchical clustering/heatmap of the compounds quantified through NMR was obtained with the software R version 4.1.2 with the packages ClassDiscovery and gplots, using the Euclidean distance and the ‘ward.D2′ clustering method.

### 3.5. Hormone Analysis

For the phytohormones levels determination, berry tissue from different time points and treatments was lyophilized before the analysis. One hundred mg of dry sample was extracted with 375 µL of methanol:isopropanol:glacial acetic acid (50:49:1, v:v:v), using vortexing and ultrasonication for 30 min. Extracts were centrifuged at 13,000 rpm for 10 min at 4 °C. After centrifugation, the supernatant was collected, and the pellet was re-extracted following the same procedure. Supernatants were pooled and filtered with 0.22 µm PTFE filters (Phenomenex, Torrance, USA) before analyses. Phytohormones, including abscisic acid (ABA), auxin indole-3-acetic acid (IAA), jasmonic acid (JA), and its conjugate jasmonoyl-isoleucine (JA-Ile), jasmonic acid precursor 12-*oxo*-phytodienoic acid (OPDA), salicylic acid (SA), cytokinins *trans*-zeatin (Z), its riboside *trans*-zeatin riboside (ZR), 2-isopentenyl adenine (2iP) and its riboside isopentenyl adenosine (IPA), gibberellins GA_1_, GA_3_, GA_4_ and GA_7_, and the precursor of ethylene 1-aminocyclopropane-1-carboxylic acid (ACC), were analyzed by UHPLC-ESI-MS/MS in MRM mode as previously described [165]. Quantification was made by calculating recovery rates for each sample by using deuterium-labeled compounds as internal standards (OlChemIm Ltd. (Olomouc, Czech Republic).

### 3.6. NMR Analysis

A total of 40 berries were analyzed separately. Pulverized samples of berry were freeze-dried overnight (−56 °C, <0.100 mbar; Alpha 1–2 LDplus, Martin Christ), yielding 200 ± 5 mg of dried powder each. Samples were then stored at −80 °C. Polar and hydrophobic metabolites were extracted with deuterated solvents by using a chloroform/water/methanol solution 10:10:1 (similarly to [166]). In detail, each sample was initially inserted in a Teflon tube, added with 0.1 mL of CD_3_OD and a 0.5 mL solution of D_2_O 0.4 M phosphate buffer (pH = 6.86), then mixed for 10 min. An aliquot of 1 mL CDC_l3_ and 0.5 mL of D_2_O phosphate buffer was then added, followed by another 10 min of mixing. Samples were then kept for 10 min on ice and then centrifuged at 5166× *g* for 15 min at 4 °C. Polar and hydrophobic phases were separated and transferred into 5 mm NMR tubes.

NMR spectra were acquired with a Bruker Avance Neo 600 MHz spectrometer (Bruker BioSpin, Karlsruhe, Germany) equipped with a Prodigy cryoprobe and using Topspin 4.1.1 software, applying a noesypr1D pulse sequence with spectral width 12 kHz, acquisition time 2.99 s, 16 scans, relaxation delay 4 s, and four dummy scans. All spectra were processed with an ACD NMR processor 12.1 (shortened to ACD, ACD Labs).

Free induction decay (FID) was multiplied by a Lorentzian broadening of 0.3 Hz before applying the Fourier transformation. Transformed spectra were further processed in ACD, performing manual phasing and automatic baseline correction via the FID reconstruction model. Peaks in the ^1^D-NMR spectra were initially identified by comparison with similar metabolomics characterizations reported in the literature [167,168,169,170,171]. A more precise approach was then adopted, submitting known chemical shifts to public databases such as the Human Metabolome DataBase [172] (HMDB), the Biological Magnetic Resonance DataBase [173] (BMRB), and nmrshiftdb2 [174], and checking the peak relative intensity, multiplicity, and J coupling for each proton of each identified compound. Some uncertain assignments were confirmed with spiking experiments and by ^2^D-NMR spectra.

Spectra were grouped together, and intelligent bucketing was performed in ACD (the water suppression region was excluded). A total of 140 buckets were selected for berries spectra. Signal area values were organized into a matrix to be processed using MetaboAnalyst 5.0 (accessible at https://www.metaboanalyst.ca/ accessed on 15 December 2022, [175]).

## 4. Conclusions

The findings pointed out in the present research may impact viticulture at different levels, from the agronomic management of the vineyard to wine quality, passing through a physiological characterization of the berry response to stress.

Concerning the latter aspect, we proposed a model that summarizes the main responses of the berry to both the single and sequential stresses. This model may open the way not only to future research on grapevine stress physiology but also to its integration with existing data in order to build a broader knowledge base for one of the most economically relevant crops grown in the temperate area. Moreover, our model, pointing out a predominant well-known response to water loss and heat stress, i.e., the accumulation of osmoprotectants, may definitely offer inspiration for developing new biostimulants and chemicals aimed at stabilizing cell membranes, proteins, and metabolic pathways against the denaturing effects of stress.

Another important piece of information coming from our study is represented by the acceleration of berry ripening in the vines that underwent flooding stress at bud-break. Despite the months passed since such an extreme event, we hypothesize that a sort of plant stress memory (PSM) mechanism may affect not only the ripening behavior in flooded vines (F) but also the interaction with the summer heatwave in FH vines. Our study should be added to the already existing long list of cases recently reported by Charng et al. [176] and deserve further dedicated experiments aimed at identifying the molecular basis of these mechanisms.

Finally, some considerations must be made about the potential effects of single and sequential stresses on wine quality. One of the main issues may be represented by the high concentration of some specific amino acids, such as proline, alanine, and GABA, in the most derive from stressed vines. These amino acids may indeed affect both fermentation and the final sensory characteristics of the wine, as alanine is one of the preferential sources of nitrogen for yeasts [177], whereas proline could be the principal source of nitrogen during the maturation phase [178].

As a general remark, taken as a whole, our results may support the development of sustainable vineyard management solutions to improve the water use efficiency and adaptation capacity of actual viticultural systems to future scenarios, thus providing relevant information also for the wine-making sector.

## Data Availability

RNASeq raw data can be retrieved from the Gene Expression Omnibus (GEO) database (https://www.ncbi.nlm.nih.gov/geo/ accessed on 15 December 2022) under the accession number GSE206753.

## Supplementary Materials

The following supporting information can be downloaded at: https://www.mdpi.com/article/10.3390/plants11243574/s1, Figure S1: mean cross diameter of all berries from T1 to T5; Figure S2: validation of RNAseq expression levels in a subset of DEGs; Figure S3: expression levels of *DEWAX* gene; Figure S4: levels of hormone precursors and metabolites; Figure S5: RNAseq expression of ABA- and ethylene-related genes; Figure S6: RNASeq expression profiles of CK-related genes; Figure S7: RNASeq expression profiles of GA- and SA-related genes: Figure S8: RNAseq expression of genes found in the ABA subnetwork; Figure S9: grape bunches collected from T1 to T5; Figure S10: sugar compounds quantification through NMR; Figure S11: organic acids, amino acids and CGA quantification through NMR; Figure S12: RNASeq expression profiles of a subset of metabolic genes; Table S1: summary of the RNASeq reads statistics; Table S2: list of Differentially Expressed Genes (DEGs) in all the analyzed contrasts; Table S3: GO term enrichment of the genes belonging to SA subnetwork; Table S4: sequences of primers used in qPCR; Table S4: sequences of the primers used for qPCR.
