# Supplementary material for: Grape Berry Responses to Sequential Flooding and Heatwave Events: A Physiological, Transcriptional, and Metabolic Overview
Source: Plants (Basel). 2022 Dec 17;11(24):3574. doi: 10.3390/plants11243574 (PMC9788187; doi:10.3390/plants11243574)
Supplement: Supplementary file 1 [file plants-11-03574-s001.zip › Supplementary Figures.pdf]

1. Supplementary Figures

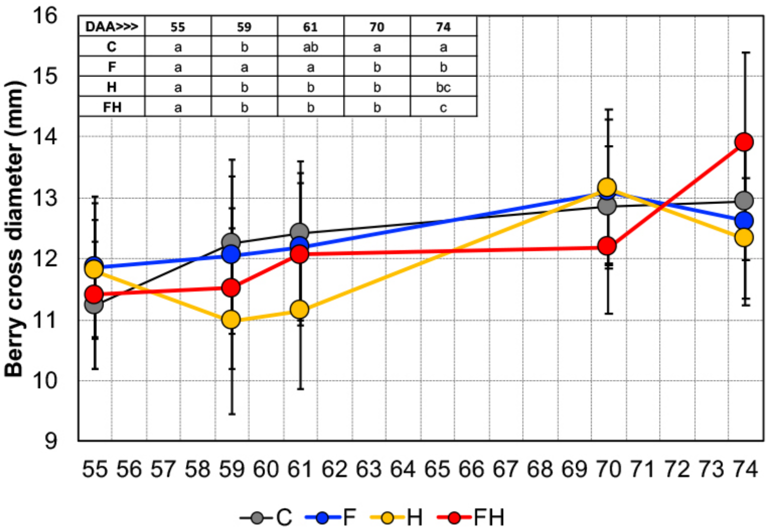

Figure S1. Cross diameter of berries from all treatments (C, control; F, flooded; H, heatwave; FH, flooded plus heatwave) measured from T1 (55 DAA) to T5 (74 DAA). Bars represent standard deviation (n ≥ 10). Statistics are show at the top of the chart with different letters representing statistically different values (P ≤ 0.05).

plants

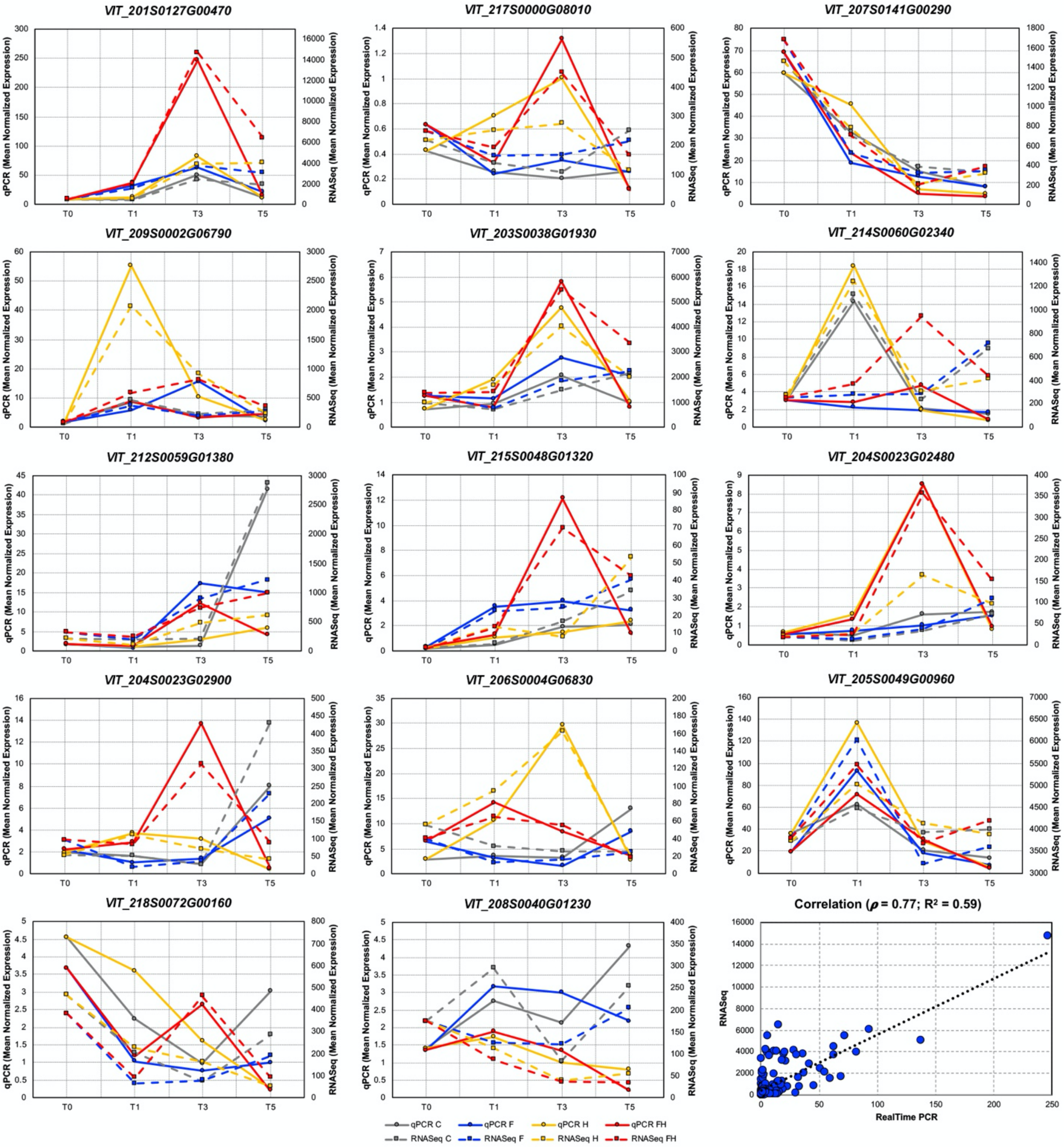

Figure S2. Validation of RNASeq expression levels (dashed lines) with qPCR (continuous lines) carried out on all samples (C, control; F, flooded; H, heatwave; FH, flooded plus heatwave) on a subset of genes chosen among the DEGs according to their different levels of expression. At the bottom right of the figure, a correlation plot is also show along with the corresponding Pearson (ρ) coefficient and the R^2.

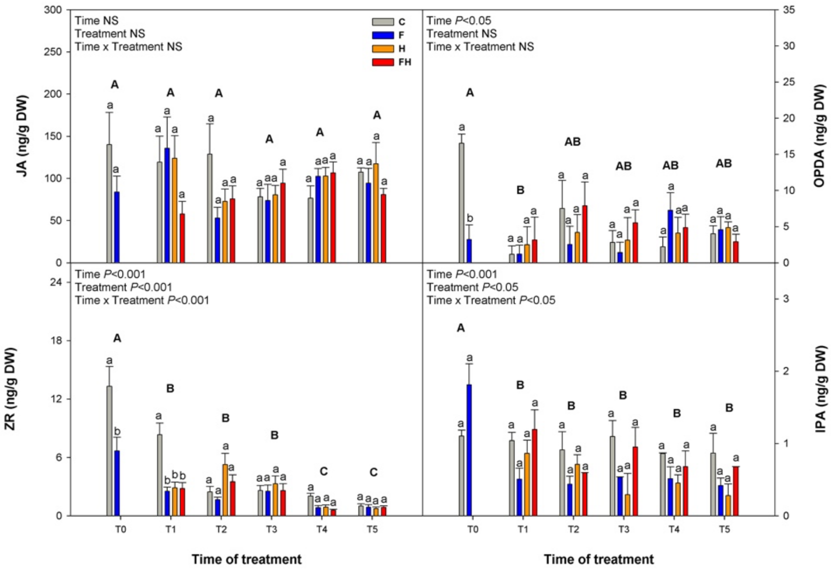

Figure S3. Levels of a subset of hormone precursors/metabolites in berries samples (C, control; F, flooded; H, heatwave; FH, flooded plus heatwave). The results of the statistical analyses are reported within each chart along with the letter indicating statistically different values (uppercase for the timepoint and lowercase for the treatments).

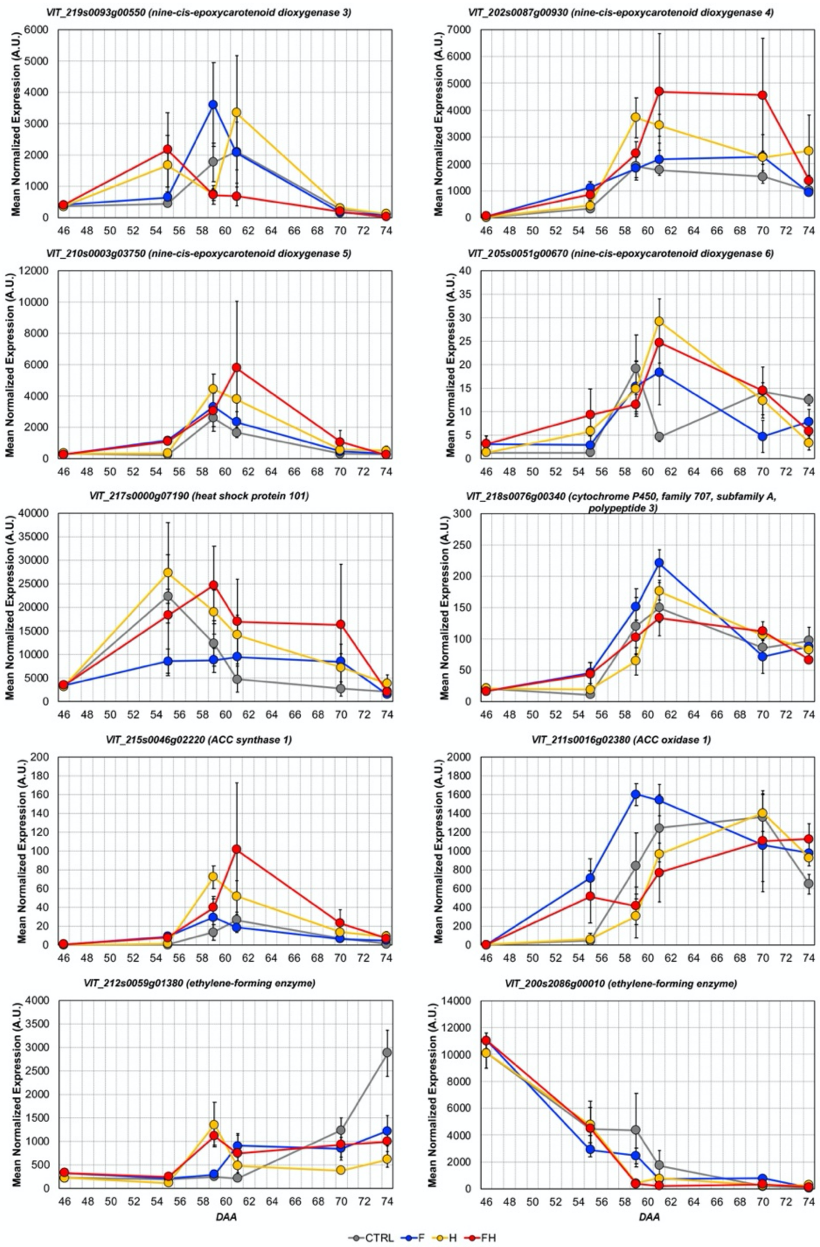

Figure S4. RNASeq expression profiles of a subset of ABA- and ethylene-related genes in all samples (C, control; F, flooded; H, heatwave; FH, flooded plus heatwave) from 46 (T0) to 74 (T5) DAA (Days After Anthesis). Bars represent standard deviation (n = 3).

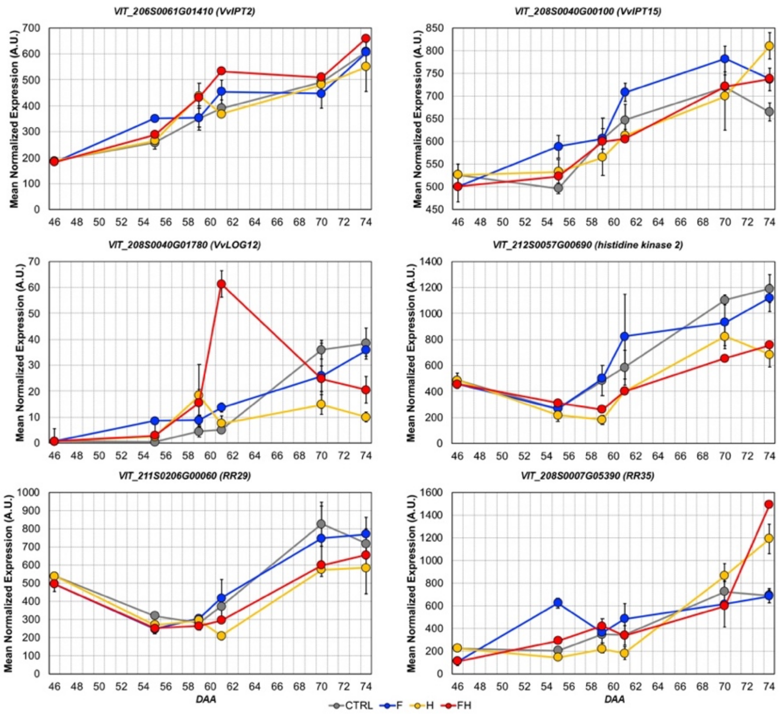

Figure S5. RNASeq expression profiles of a subset of CK-related genes in all samples (C, control; F, flooded; H, heatwave; FH, flooded plus heatwave) from 46 (T0) to 74 (T5) DAA (Days After Anthesis). Genes were chosen according to the study carried out by Böttcher et al. (2015). Bars represent standard deviation (n = 3).

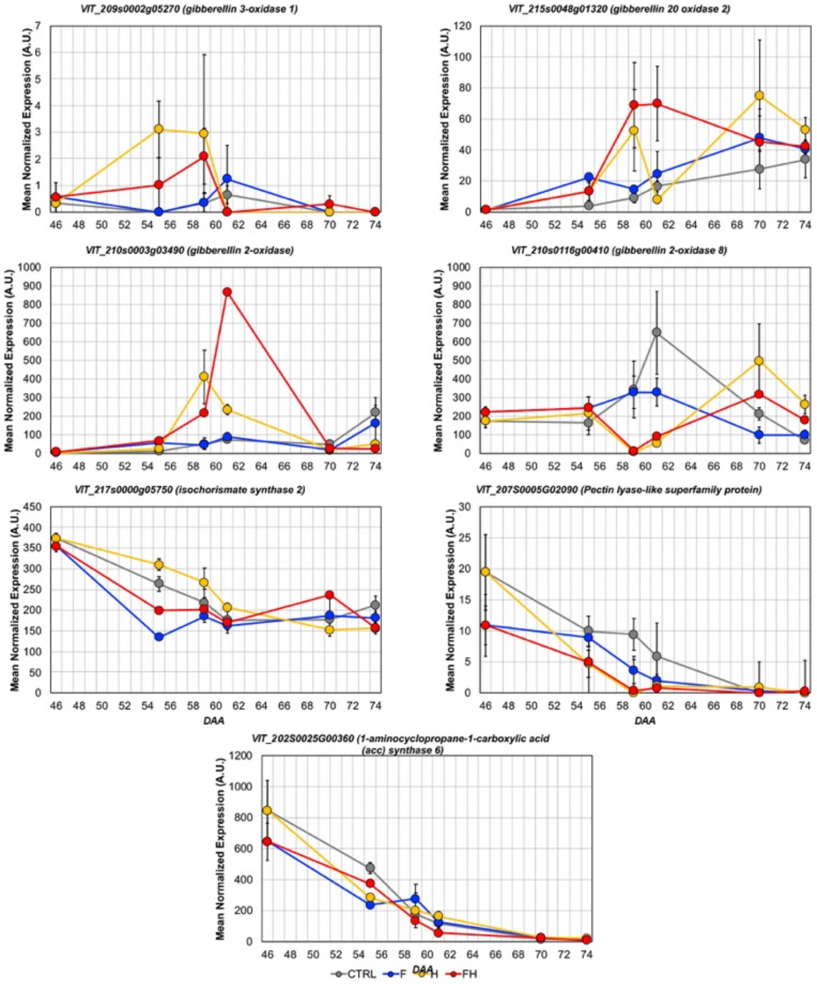

Figure S6. RNASeq expression profiles of a subset of GA- and SA-related genes in all samples (C, control; F, flooded; H, heatwave; FH, flooded plus heatwave) from 46 (T0) to 74 (T5) DAA (Days After Anthesis). Bars represent standard deviation (n = 3).

plants

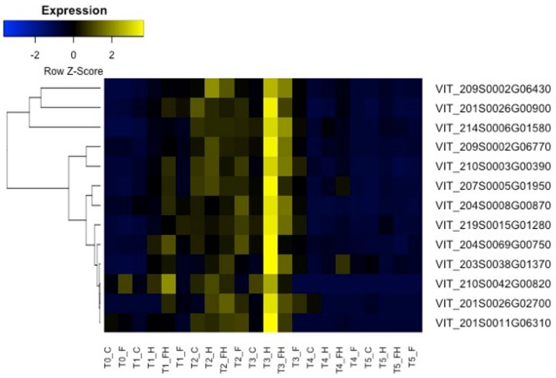

Figure S7. Heatmap of RNAseq expression of genes found in the ABA subnetwork.

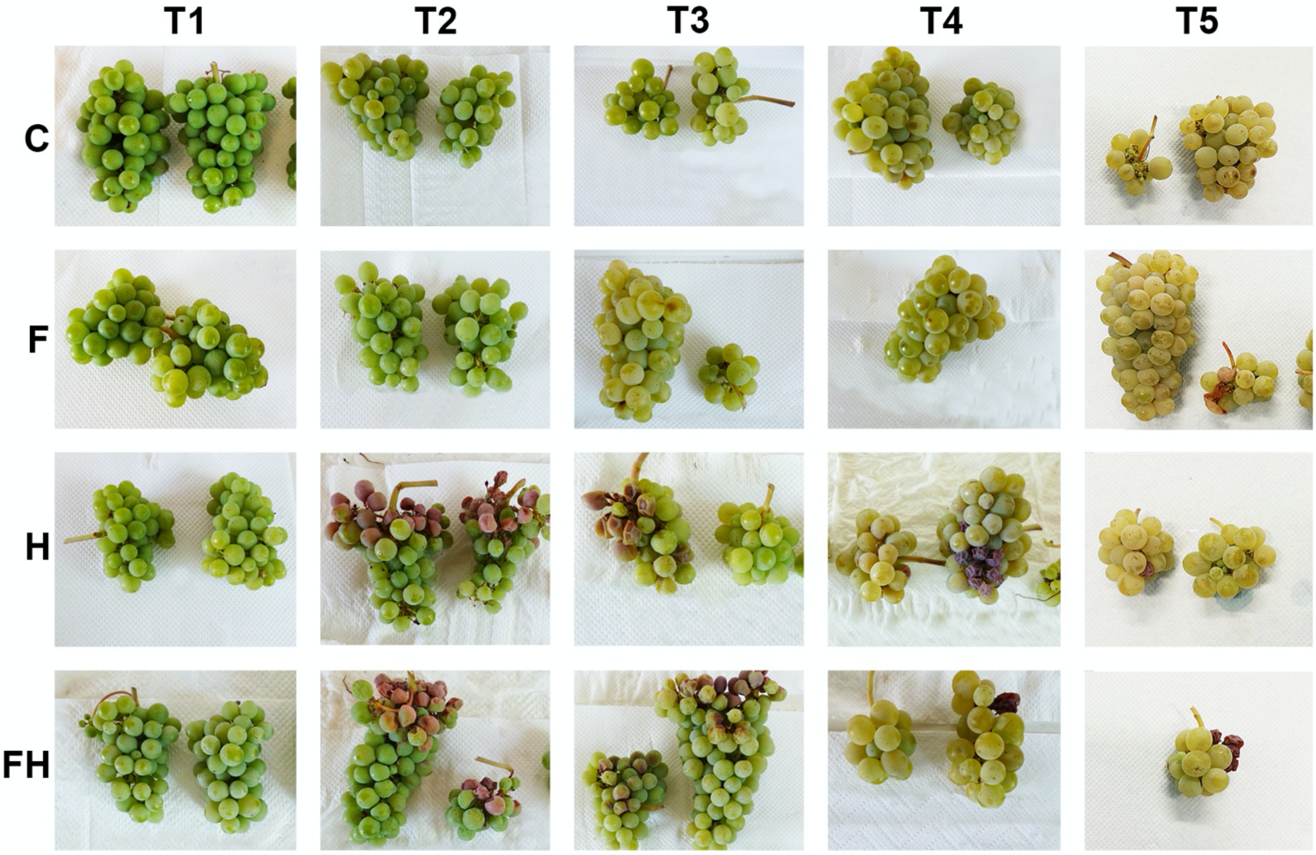

Figure S8. Samples of grape bunches collected from 55 (T1) to 74 (T5) DAA from all the treated vines (C, control; F, flooded; H, heatwave; FH, flooded plus heatwave).

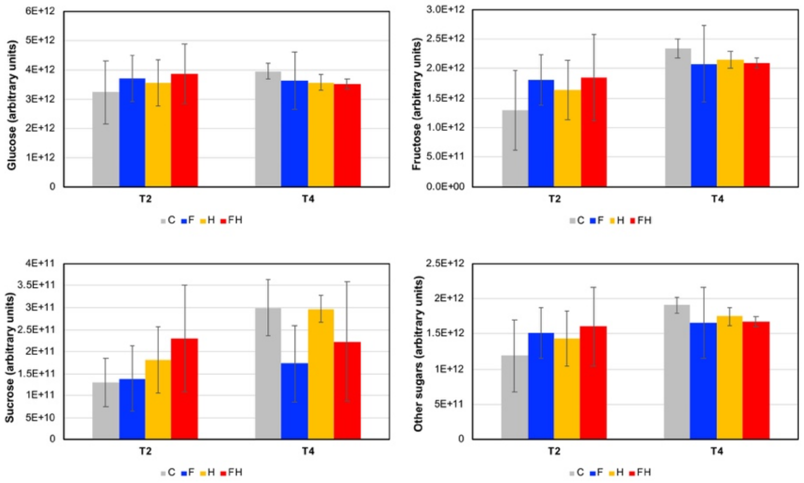

Figure S9. Quantification at T2 and T4 (C, control; F, flooded; H, heatwave; FH, flooded plus heatwave) of the sugars identified (glucose, fructose and sucrose) or non identified (other sugars) through NMR. Bars represent standard deviation (n = 5).

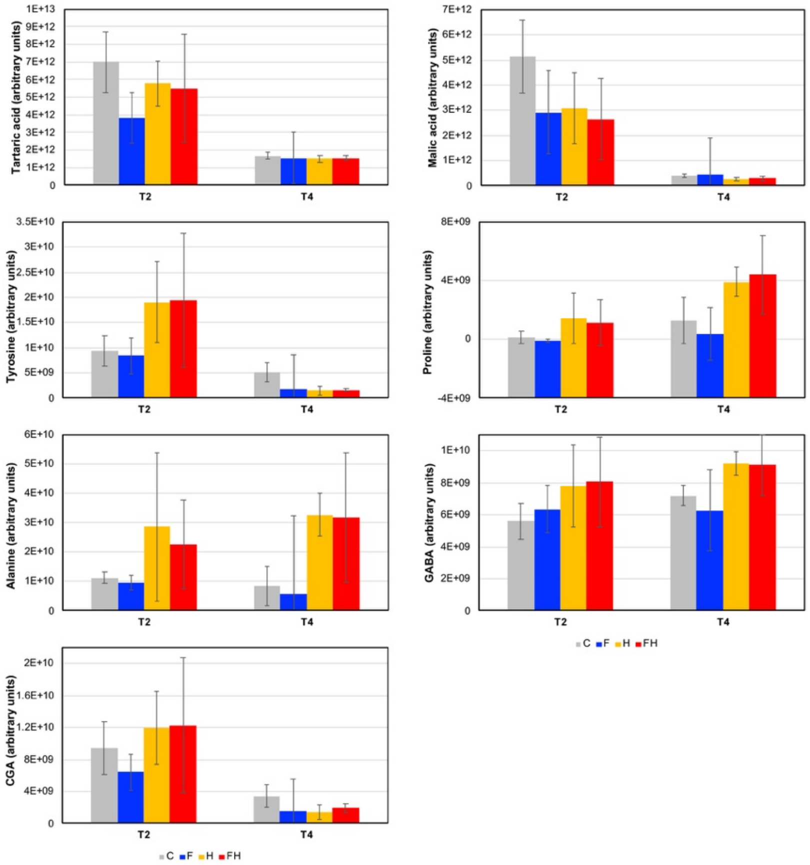

Figure S10. NMR quantification at T2 and T4 (C, control; F, flooded; H, heatwave; FH, flooded plus heatwave) of a subset of compounds including organic acids (tartaric and malic acid), amino acids (tyrosine, proline, alanine and GABA) and chlorogenic acid (CGA). Bars represent standard deviation (n = 5).

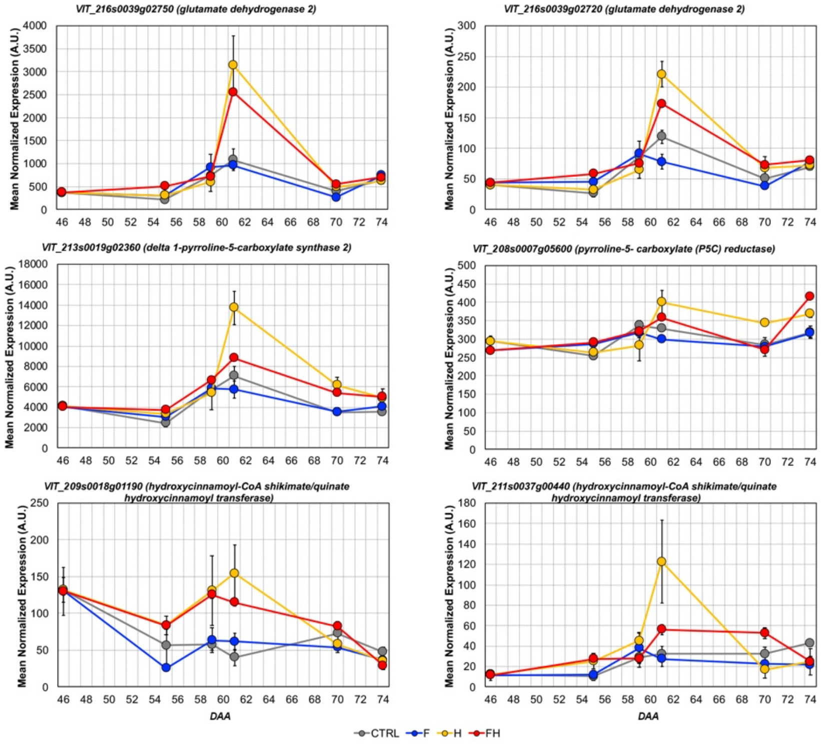

Figure S11. RNASeq expression profiles of a subset of metabolic genes in all samples (C, control; F, flooded; H, heatwave; FH, flooded plus heatwave) from 46 (T0) to 74 (T5) DAA (Days After Anthesis). Bars represent standard deviation (n = 3).
